# The Diverse Effect of HDAC Inhibitors: Sodium Butyrate and Givinostat on Microglia Polarization After Hypoxia-Ischemia In Vitro

**DOI:** 10.3390/ijms27021114

**Published:** 2026-01-22

**Authors:** Karolina Ziabska, Paulina Pawelec, Luiza Stanaszek, Malgorzata Ziemka-Nalecz

**Affiliations:** NeuroRepair Department, Mossakowski Medical Research Institute, Polish Academy of Sciences, 5 A. Pawinskiego Str., 02-106 Warsaw, Poland; kziabska@imdik.pan.pl (K.Z.); ppawelec@imdik.pan.pl (P.P.); lstanaszek@imdik.pan.pl (L.S.)

**Keywords:** oxygen and glucose deprivation (OGD), histone deacetylase inhibitor (HDACi), sodium butyrate (SB), Givinostat (ITF2357), BV2 microglia, M1/M2 phenotype, PI3K/AKT

## Abstract

Microglia play a key role in the development of neuroinflammation induced by cerebral ischemia. On the other hand, these cells participate in neurorepair processes. This dual role of microglia stems from the ability to shift their phenotype from pro-inflammatory M1 to protective M2. Histone deacetylase inhibitors (HDACis) are a group of agents that exhibit neuroprotective effects in some models of ischemia, among others, by modulation of signaling pathways that regulate microglial activation. This study aimed to examine the effect of HDACis—sodium butyrate and Givinostat—on polarization of microglia and their potential mechanism of action in a model of ischemia in vitro (oxygen and glucose deprivation, OGD). We examined the expression of pro- and anti-inflammatory markers in the BV2 microglial cell line after OGD and HDACis treatment by qPCR; polarization of microglia by flow cytometry; and the activation/phosphorylation of ERK and AKT in BV2 cells by Western blot and ELISA. Our findings demonstrate a divergent impact of HDACis on the phenotype of microglial cells. Sodium butyrate significantly suppressed the mRNA expression of pro-inflammatory markers (IL-1β, TNF-α, CD86) and increased the level of anti-inflammatory factors in BV2 microglial cells after OGD, whereas Givinostat failed to attenuate these inflammatory responses. Our findings demonstrate that sodium butyrate, but not Givinostat, promotes a shift in microglia toward an anti-inflammatory M2 phenotype under ischemic conditions. This effect is associated with suppression of pro-inflammatory gene expression and activation of the PI3K/AKT signaling pathway. These results identify sodium butyrate as a potential modulator of microglial responses following ischemic injury.

## 1. Introduction

Microglia are the resident immune cells of the central nervous system (CNS), not only playing a key role in maintaining homeostasis in the brain tissue but also responsible for the development of inflammation after CNS injuries, including cerebral ischemia. Under physiological conditions, microglia possess numerous thin and highly branched processes that dynamically extend and retract to survey the surrounding environment for potential pathogens or disturbances. Following ischemic injury, microglia become rapidly activated, undergo a morphological transformation into an amoeboid form, and acquire the ability to migrate, proliferate, and phagocytose [1,2]. Activated microglia may play both beneficial and detrimental roles following injury, with the outcome depending on the specific activation phenotype. Their response may be shifted toward the release of the pro-inflammatory mediators that exacerbate neuroinflammation and contribute to secondary brain injury after ischemia [3,4], or alternatively toward the anti-inflammatory phenotype that supports neurorepair processes [5,6].

Microglia exhibit the capacity for polarization—the ability to alter their phenotype in response to various external stimuli, including cytokines and lipopolysaccharide (LPS). This phenotypic shift is accompanied by profound changes in the cellular proteome, leading to the expression of specific surface markers characteristic of distinct activation states. Under in vitro conditions, depending on the applied stimulus, microglia can adopt a classically activated M1 phenotype or alternatively activated M2 phenotype [7,8]. A key feature of microglial physiology is their phenotypic plasticity, enabling a dynamic transition between the M1 (pro-inflammatory) and M2 (anti-inflammatory or reparative) states in response to microenvironmental cues. M1-polarized microglia secrete pro-inflammatory cytokines (e.g., IL-1β, IL-2, IL-6, IL-12, IL-18, IL-23; TNF-α), COX-2, and metalloproteinases (MMPs) that compromise the integrity of the blood-brain barrier (BBB), thereby promoting leukocyte infiltration into the central nervous system. Infiltrating immune cells, in turn, release additional pro-inflammatory mediators and reactive oxygen and nitrogen species, exacerbating neuronal damage [9,10]. In contrast, M2-polarized microglia produce trophic factors (NGF, BDNF, GDNF, TGF-β) and anti-inflammatory cytokines (IL-4, IL-10), which attenuate macrophage accumulation in the injured brain tissue and activate cellular mechanisms involved in repair and regeneration [11,12]. Many studies indicate that complete inhibition of microglial activation following ischemic injury is not beneficial, as it may lead to exacerbated brain damage [13,14]. Current researches are focused on modulating microglial activity in order to promote the predominance of the M2 phenotype, which is believed to exert neuroprotective and reparative effects within the post-ischemic brain.

Accumulating data indicate that post-translational modifications of chromatin (epigenetic modification) have profound effects on gene expression and are responsible for regulating critical intracellular pathways. One of the types of chromatin modification is the acetylation and deacetylation mediated by histone acetylases (HATs) and histone deacetylases (HDACs) [15]. In general, HATs acetylate lysine residues within the N-terminal tails of histone proteins, causing a more relaxed chromatin structure and promoting transcriptional activation. Conversely, histone deacetylases (HDACs) remove acetyl groups from histones, leading to chromatin condensation and suppression of transcription. Moreover, other non-histone proteins (e.g., transcription factors, signal transduction mediators, cytoskeletal proteins) are also modified by HDACs/HATs, which regulate their interaction, localization, and stability [16]. There is growing evidence that epigenetic modifications have been associated with the pathogenesis of cerebral ischemia. After ischemic insult, aberrant HDAC activity contributes to neuronal death, neuroinflammation, and disruption of cellular homeostasis. Several data provided evidence that histone deacetylase inhibitors (HDACis) are a promising group of agents that demonstrate neuroprotective and anti-inflammatory action in animal models of brain ischemia, and these beneficial effects are related to modulating microglial activation and polarization [17,18]. The effect of HDACis on M1/M2 microglial polarization has not yet been fully elucidated despite several in vitro studies over the last few years. Treatment with HDAC inhibitors such as valproic acid (VPA) and sodium butyrate (SB) has been shown to alter both pro-inflammatory (M1) and anti-inflammatory (M2) activation states in LPS-stimulated rat microglia [19]. Conversely, pre-treatment with trichostatin A (TSA) was found to suppress not only LPS-induced inflammatory responses but also IL-4–induced mRNA expression of M2 phenotype markers [20]. These observations imply that HDAC inhibition may promote microglial polarization into an anti-inflammatory phenotype. Also, our previous studies have shown that SB treatment after neonatal hypoxia-ischemia (HI) increased the number of polarized M2 phenotype cells with a concomitant decrease of pro-inflammatory cytokines [17,21].

The molecular mechanism of HDAC inhibitors associated with the alteration of microglial polarization following ischemia is not fully elucidated. Recent evidence suggests that HDAC inhibitors can modulate microglial polarization through epigenetic and non-epigenetic mechanisms [22,23]. Besides their classical role in histone acetylation, HDACis have been shown to influence key intracellular signaling cascades that determine the microglial activation state. In particular, modulation of the PI3K/AKT and MAPK pathways appears to play a central role in shifting microglia from a pro-inflammatory M1 phenotype toward an anti-inflammatory/neuroprotective M2 phenotype [18]. Activation of PI3K/AKT signaling promotes the expression of anti-inflammatory mediators and enhances cellular survival pathways, while regulation of MAPK signaling (including ERK) contributes to the suppression of pro-inflammatory gene expression.

In the present study, we investigated the effect of HDAC inhibitors on the expression profile of major pro- and anti-inflammatory genes and polarization of microglia BV2 cell line in a model of OGD, an established model of hypoxia-ischemia in vitro. Moreover, we also examine the influence of HDACis on PI3K/AKT and MAPK/ERK signaling pathways in microglial cells subjected to ischemic conditions in vitro. As the effect of histone deacetylase inhibition on microglia response after ischemic injury may be agent-dependent, we have investigated the action of sodium butyrate (SB), a short-chain fatty acid, as well as another deacetylase inhibitor—Givinostat (ITF2357), a derivative of hydroxamic acid, thus presenting a different chemical structure than SB. Sodium butyrate, beyond its epigenetic effects, has been shown to modulate intracellular signaling pathways involved in cell survival and inflammation, including activation of the PI3K/AKT pathway and regulation of MAPK/ERK signaling, which are critical for microglial polarization and functional responses [24,25]. Givinostat is a pan-HDAC inhibitor with strong anti-inflammatory activity, originally developed for the treatment of inflammatory and neuromuscular disorders. Previous studies indicate that Givinostat suppresses the expression of pro-inflammatory mediators; however, the effects of this HDAC inhibitor on microglial polarization and the involvement of PI3K/AKT and MAPK signaling pathways under ischemic conditions remain poorly characterized [26,27].

## 2. Results

### 2.1. The Effect of HDAC Inhibitors on Polarization of BV2 Microglia—qPCR Studies

In the first step of the study, quantitative PCR analysis was used to evaluate the effect of HDAC inhibitors—SB (in concentration 1 mM) and Givinostat (in concentration 1 μM) on the expression of pro- and anti-inflammatory genes in microglial BV2 cells in control cultures as well as in cultures subjected to the OGD procedure. The experiments were conducted at 24 and 72 h after OGD and in time-matched control cultures. In the qPCR analyses, we examined the mRNA expression of markers characteristic of M1 microglia (IL-1β, TNF-α, and CD86) and M2 microglia (IL-4, arginase-1, and CD206). Although these markers represent different functional categories: surface receptors (CD86, CD206), secreted cytokines (IL-1β, TNF-α, IL4), and intracellular enzymes (arginase-1), they collectively reflect the activation and polarization status of microglia and are therefore frequently analyzed together in polarization studies.

**Figure 1:**
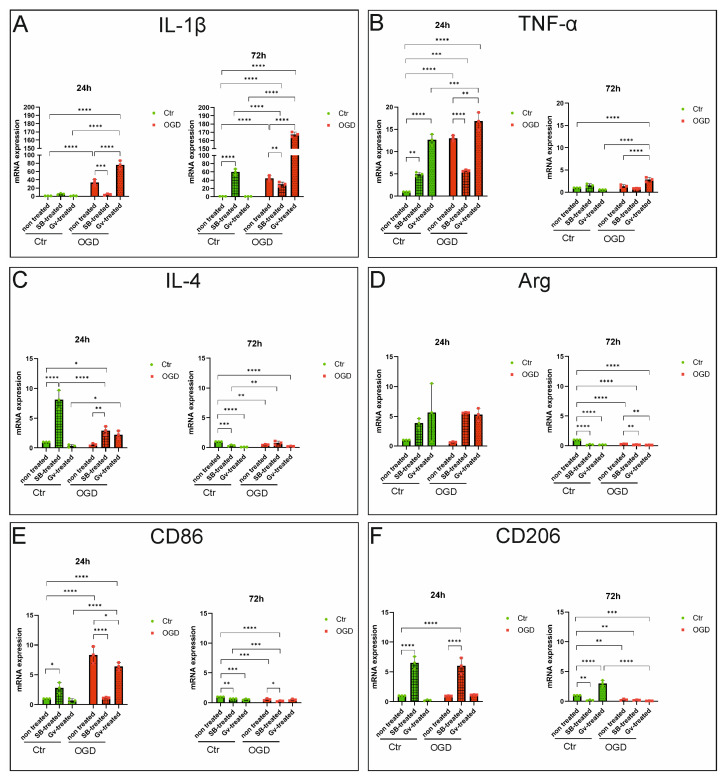
Expression of the mRNA of proteins characteristic of the pro- and anti-inflammatory profile of microglia. A qPCR analysis was performed on material derived from BV2 microglia cultures that underwent the OGD procedure or control cells, in the presence or absence of sodium butyrate (in concentration 1 mM) or Givinostat (in concentration 1 μM). The material was collected 24 h and 72 h after the OGD procedure. The analysis of changes in the mRNA level of genes was carried out using SYBR™ Green PCR Master Mix Reagent (Thermo Fisher Scientific, Foster City, CA, USA). The quantitative PCR reactions were performed in the 7500 Fast Real-Time PCR System (Applied Biosystems, Foster City, CA, USA). Each sample was tested in triplicate during two analysis sessions. The dissociation curve will be plotted to determine the specificity of the amplification. The fluorescence signals of a specific transcript were normalized against those of the reference gene (*β-actin*), and the threshold cycle values (ΔCt) were quantified as fold changes using the 2^−ΔΔCT^ method. Detailed information about the methodology and starters used can be found in Section 4.2 in the Materials and Methods Section. (**A**)—analysis results for *IL-1β*, (**B**)—analysis results for *TNF-α*, (**C**)—analysis results for *IL-4*, (**D**)—analysis results for arginase, (**E**)—analysis results for *CD86*, (**F**)—analysis results for *CD206*. Ctr—control, OGD—oxygen-glucose deprivation. Results are presented as mean values ± standard deviation (SD), calculated relative to the β-actin reference gene. Color green—control, color red—OGD. Number of biological replicates in each experimental group n = 3. Statistical analysis: two-way ANOVA with Bonferroni post-hoc test: **** *p* < 0.0001, *** *p* < 0.001, ** *p* < 0.01, * *p* < 0.05.

The mRNA expression levels of the evaluated pro-inflammatory cytokines (IL-1β, TNF-α) and anti-inflammatory proteins (IL-4, arginase), as well as mRNA of the surface markers of the M1 and M2 phenotypes (CD86 and CD206, respectively), were calculated relative to the reference gene for β-actin, which was amplified from the same cDNA. Analysis of the obtained results showed increased mRNA expression of all pro-inflammatory genes 24 h after OGD procedure compared to the control, non-treated samples (for *IL-1β*, *p*-value < 0.0001; *TNF-α*, *p*-value < 0.0001, *CD86*, *p*-value < 0.0001) (Figure 1A,B,E). Supplementation of culture medium with 1 mM SB for 24 h following hypoxia-ischemia significantly reduced the mRNA level of *IL-1β* (*p*-value = 0.0002), *TNF-α* (*p*-value < 0.0001), and *CD86* (*p*-value < 0.0001) in BV2 cells, to the level observed in control cultures (Figure 1A,B,E). The use of the Givinostat after OGD did not decrease the expression of *IL-1β* and *TNF-α*. On the contrary, we observed an additional increase in expression of these cytokines after the Givinostat application 24 h after OGD (for *IL-1β*, *p*-value < 0.0001; *TNF-α*, *p*-value = 0.0014) (Figure 1A,B). Givinostat only slightly reduced the expression of CD86 24 h after OGD (*p*-value = 0.0321) (Figure 1E). A similar pattern of the pro-inflammatory markers expression after OGD and HDACis was observed at the second investigated time point. The level of *IL-1β* significantly increased 72 h after OGD (*p*-value < 0.0001), and although sodium butyrate reduced this expression, the effect was less pronounced than at the earlier time point (*p*-value = 0.0077). Oppositely, the use of Givinostat after ODG resulted in a fourfold increase in *IL-1β* expression in BV2 cells compared to the cultures not exposed to this inhibitor (*p*-value < 0.0001) (Figure 1A). Expression of the *TNF-α* in microglial cells 72 h after OGD did not change significantly compared to the control culture (not subjected to the OGD procedure) (*p*-value > 0.9999) (Figure 1B). Enhanced expression of *TNF-α* mRNA was observed only in the OGD+Givinostat experimental group (*p*-value < 0.0001) (Figure 1B). The level of *CD86* significantly decreased 72 h after OGD (*p*-value = 0.0003), and although sodium butyrate reduced this expression (*p*-value = 0.0175). It is worth noting that the presence of HDAC inhibitors in control BV2 cultures can enhance the expression of pro-inflammatory markers, particularly *TNF-α* (24 h after treatment with SB, *p*-value = 0.0011; 24 h after treatment with Givinostat, *p*-value < 0.0001).

Oxygen glucose deprivation did not influence the expression of anti-inflammatory markers (*IL-4*, arginase, and *CD206*) in the BV2 cells at the first investigated time point (24 h) compared to the control culture (*p*-values > 0.9999) (Figure 1C,D,F). Extension of the cell culturing to 72 h after OGD resulted in a small but statistically significant reduction in the expression of all investigated anti-inflammatory markers in BV2 cells subjected to OGD (for *IL-4*, *p*-value = 0.0015; for arginase, *p*-value < 0.0001; for *CD206*, *p*-value = 0.0063). Treatment with SB markedly enhanced the expression of anti-inflammatory markers *IL-4* and *CD200*, both under control conditions and following OGD at the earlier time point (24 h) (for *IL-4* under control conditions, *p*-value < 0.0001, following OGD, *p*-value = 0.0095, for arginase under control conditions, *p*-value = 0.9251, following OGD, *p*-value = 0.055, for *CD206* under control conditions, *p*-value < 0.0001, following OGD, *p*-value < 0.0001). However, after extending the culture period to 72 h, this effect was no longer observed in BV2 cells. Treatment with SB decreased the expression of the anti-inflammatory markers under control conditions (for IL-4, *p*-value = 0.0002; for arginase, *p*-value < 0.0001; for CD206, *p*-value = 0.0023). In contrast, Givinostat treatment led to a statistically significant increase only in *CD206* expression under control conditions at the later time point (72 h) (*p*-value < 0.0001). Notably, Givinostat did not statistically significantly increase the expression of anti-inflammatory markers at any time point following OGD (the range of *p*-values was from 0.0826 to above 0.9999) (Figure 1C,D,F). Contrarily, we observed a modest but statistically significant decrease in arginase expression 72 h after OGD compared with the OGD group not treated with this inhibitor (OGD+Gv vs. OGD) (*p*-value = 0.001).

### 2.2. The Effect of HDAC Inhibitors on Polarization of BV2 Microglia—Flow Cytometry Analysis

To evaluate the effect of HDAC inhibitors on microglia polarization, we measured the expression of CD86 and CD206 in control and OGD-subjected BV2 cells via flow cytometry.

**Figure 2:**
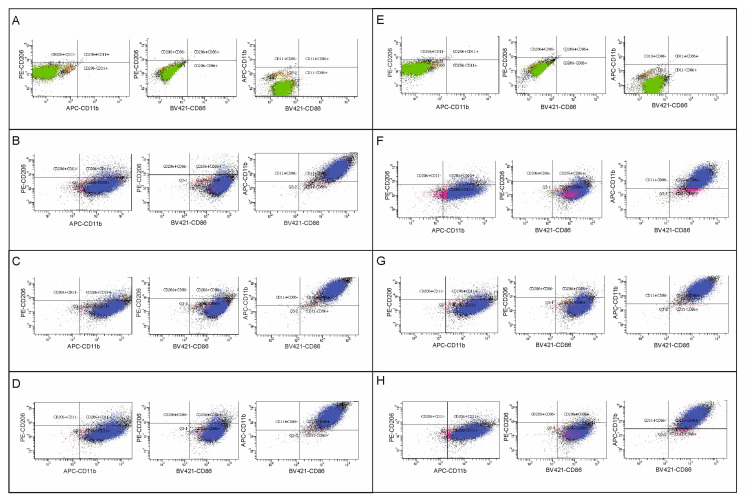
Analysis of BV2 microglial cell subpopulations—24 h after OGD. Flow cytometry analysis was performed on cell material to assess the population sizes of individual microglial phenotypes after OGD and in control microglia, in the presence or absence of sodium butyrate (in concentration 1 mM) or Givinostat (in concentration 1 μM). The material was collected 24 h after the OGD procedure. Detailed information about the methodology and antibodies used can be found in Section 4.3 in the Materials and Methods Section. (**A**–**H**)—gating strategy to identify individual microglial populations and gating results; (**A**)—unlabeled control microglia; (**B**)—labeled control microglia; (**C**)—labeled control microglia treated with sodium butyrate; (**D**)—labeled control microglia treated with Givinostat; (**E**)—unlabeled microglia after OGD; (**F**)—labeled microglia after OGD; (**G**)—labeled microglia after OGD, treated with sodium butyrate; (**H**)—labeled microglia after OGD, treated with Givinostat. CD11b+CD86+ cells were defined as pro-inflammatory (M1) phenotype, and CD11b+CD206+ cells as anti-inflammatory (M2) phenotype. The number of biological replicates in each experimental group was n_1_ = 4. The number of events analyzed in each experimental group was n_2_ = 30,000. The panel shows the analysis for one representative biological replicate.

**Figure 3:**
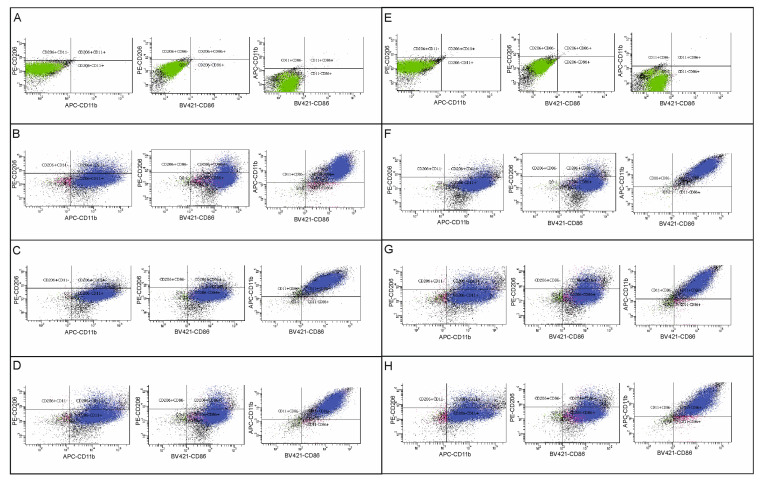
Analysis of BV2 microglial cell subpopulations—72 h after OGD. Flow cytometry analysis was performed on cell material to assess the population sizes of individual microglial phenotypes after OGD and in control microglia, in the presence or absence of sodium butyrate (in concentration 1 mM) or Givinostat (in concentration 1 μM). The material was collected 72 h after the OGD procedure. Detailed information about the methodology and antibodies used can be found in Section 4.3 in the Materials and Methods Section. (**A**–**H**)—gating strategy to identify individual microglial populations and gating results; (**A**)—unlabeled control microglia; (**B**)—labeled control microglia; (**C**)—labeled control microglia treated with sodium butyrate; (**D**)—labeled control microglia treated with Givinostat; (**E**)—unlabeled microglia after OGD; (**F**)—labeled microglia after OGD; (**G**)—labeled microglia after OGD, treated with sodium butyrate; (**H**)—labeled microglia after OGD, treated with Givinostat. CD11b+CD86+ cells were defined as pro-inflammatory (M1) phenotype, and CD11b+CD206+ cells as anti-inflammatory (M2) phenotype. The number of biological replicates in each experimental group was n_1_ = 4. The number of events analyzed in each experimental group was n_2_ = 30,000. The panel shows the analysis for one representative biological replicate.

**Figure 4:**
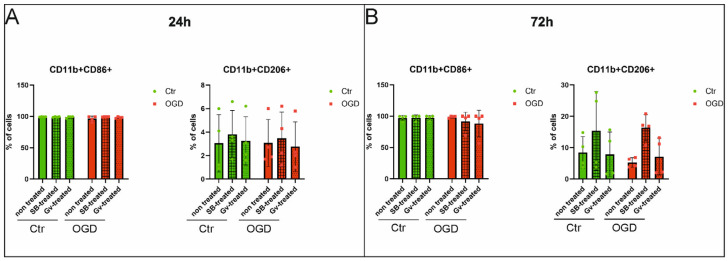
Analysis of BV2 microglial cell subpopulations. Graphs showing the calculated percentage of CD11b+CD86+, CD11b+CD206+ cells in the microglial population after OGD and control microglia, treated and untreated with sodium butyrate (in concentration 1 mM) or Givinostat (in concentration 1 μM). Material was collected 24 and 72 h after the OGD procedure. CD11b+CD86+ cells were defined as pro-inflammatory phenotype, and CD11b+CD206+ cells as anti-inflammatory. Detailed information about the methodology and antibodies used can be found in Section 4.3 in the Materials and Methods Section. (**A**)—analysis results for cells collected 24 h after OGD, (**B**)—analysis results for cells collected 72 h after OGD. Ctr—control cells, OGD—cells after the OGD procedure. Color green—control, color red—OGD. Number of biological replicates in each experimental group n_1_ = 4. Number of events analyzed in each experimental group n_2_ = 30,000. Results are presented as mean values ± standard deviation (SD). Statistical analysis: two-way ANOVA with Bonferroni post-hoc test.

Microglia BV2 cells were determined by CD11b expression. The CD11b+ population was considered to be microglia cells. Then, microglia phenotypes were determined by the expression of antibodies against the CD86 marker or the CD206 marker. The pro-inflammatory microglia population was defined as CD11b+CD86+, and the anti-inflammatory microglia population as CD11b+CD206+ (Figure 2A–H and Figure 3A–H). A list of the percentages of each population in the analyzed samples is presented in Table 1 and Table 2. The obtained results showed that control cells expressed the CD86 marker at both analyzed time points, with no significant differences in its expression between the experimental groups after OGD (the range of *p*-values for these comparisons was from 0.2121 to above 0.9999) (Figure 4A,B). Similarly, no differences in the percentage of the anti-inflammatory population were observed between groups 24 h after OGD (*p*-values > 0.9999) (Figure 4A). However, 72 h after the OGD procedure, a decrease in the CD11b+CD206+ population was noted compared to the control group (however, *p*-value > 0.9999). Administration of sodium butyrate after OGD increased the percentage of the anti-inflammatory population in the analyzed samples (*p*-value = 0.2664). Additionally, an increase in the percentage of the CD11b+CD206+ population was observed in the control group treated with SB compared to the untreated control group (*p*-value > 0.9999) (Figure 4B). However, none of the results described are statistically significant. It is noteworthy that a notable percentage of cells expressing both markers (CD86+CD206+ cells) was noted, which may suggest that some microglia are in a “transitional”/”intermediate” state between a pro- and anti-inflammatory phenotype (Table 1 and Table 2).

### 2.3. Effects of HDAC Inhibitors on Selected Signaling Pathways in Microglia After OGD Procedure—Western Blot Analyses

**Figure 5:**
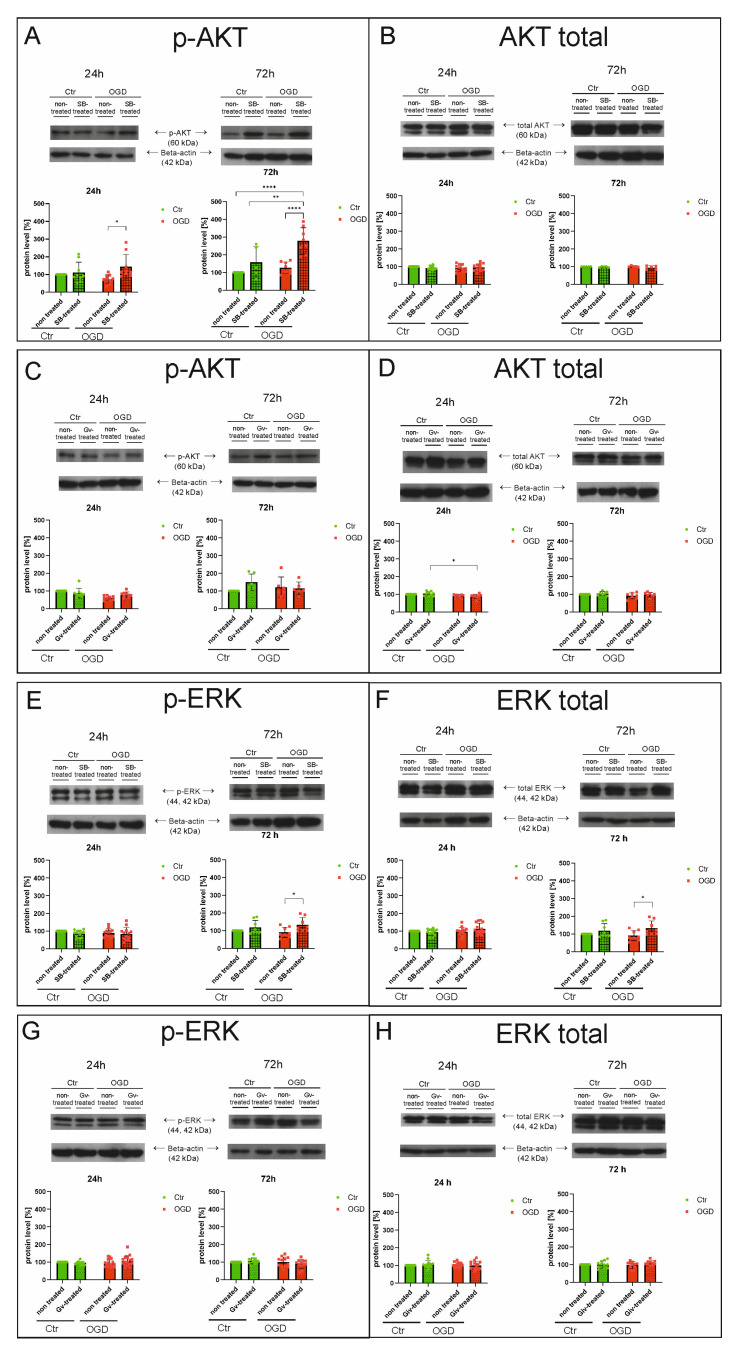
The effect of the histone deacetylase inhibitors on selected signaling pathways after the OGD procedure—Western blot analysis. Western blot analysis was performed on material derived from BV2 microglial cell cultures that underwent the OGD procedure or control cells, in the presence and absence of sodium butyrate (in concentration 1 mM) or Givinostat (in concentration 1 μM). The material was obtained 24 h and 72 h after the OGD procedure. Samples were prepared with a denaturing buffer containing β-mercaptoethanol (Bio-Rad Laboratories, Hercules, CA, USA). Total protein concentrations were assessed using a Bio-Rad DCTM protein assay kit (Bio-Rad). Samples containing 50 µg of protein were separated by SDS–PAGE electrophoresis on a 10% polyacrylamide gel at a constant voltage of 150 V for approximately 75 min. Each sample was tested at a minimum in duplicate, and each variant in three biological replicates. After electrophoresis, the proteins were transferred from the polyacrylamide gel to nitrocellulose (Protran^TM^ Supported 0.45 μm NC, Amersham Biosciences, Little Chalfont, UK) at a constant voltage of 100 V for approximately 90 min. The proteins on the nitrocellulose membrane were then subjected to immunochemical analysis with the appropriate primary antibody. The results were obtained by densitometric analysis of the gray levels of the bands obtained after electrophoretic separation, expressed as the percentage of the optical density (OD) ratio of the control SB-treated, control Gv-treated, OGD non-treated, OGD SB-treated, OGD Gv-treated bands relative to the control non-treated. A semiquantitative estimation of protein levels detected by immunoblotting was performed utilizing the LKB Utrascan XL v. 2.1 Program GelScan software. The densitometry values were averaged in all groups, and then the densitometry values in the control groups were taken as 100%. The data from the respective experimental groups are presented as percentages of the control value. Detailed information about the methodology and antibodies used can be found in Section 4.4 in the Materials and Methods Section. (**A**,**C**)—analysis results for p-AKT; (**B**,**D**)—analysis results for total AKT; (**E**,**G**)—analysis results for p-ERK; (**F**,**H**)—analysis results for total ERK. Ctr—control, OGD—oxygen-glucose deprivation. Color green—controls, color red—OGD. The results are presented as mean values ± standard deviation (SD). Number of biological replicates in each experimental group n = 3. Statistical analysis: two-way ANOVA with Bonferroni post hoc test: **** *p* < 0.001, ** *p* < 0.01, * *p* < 0.05.

The next step focused on determining the deacetylase inhibitors’ effects on selected signaling pathways involved in microglia activation. For this purpose, Western blot analyses were performed on cell lysates of BV2 microglia cultures exposed to the OGD procedure, in the presence of SB (in concentration 1 mM) or Givinostat (in concentration 1 μM), in the absence of inhibitors, and on control cultures. Material was collected 24 and 72 h after the OGD procedure. There was a decrease in the level of the phosphorylated form of AKT (p-AKT) at 24 h after the OGD procedure compared to the control culture, although this result is not statistically significant (*p*-value > 0.9999). Administration of SB after OGD increased the level of p-AKT at both investigated time points (OGD vs. OGD+SB) (for 24 h, *p*-value = 0.0124; for 72 h, *p*-value < 0.0001) (Figure 5A). The administration of Givinostat did not cause any statistically significant differences in the levels of p-AKT, compared to cells after the OGD procedure without inhibitor administration (for 24 h, *p*-value = 0.1345; for 72 h, *p*-value > 0.9999) (Figure 5C). No significant changes were noted in the levels of AKT total protein between the study groups at both time points analyzed (the range of *p*-values was from 0.1057 to above 0.9999) (Figure 5B,D), except for a decrease in the level of this protein after 24 h in the OGD-treated group, which was administered Givinostat compared to the control sample, which was administered this inhibitor (*p*-value = 0.0131) (Figure 5D).

For ERK protein, no statistically significant changes in the levels of both the phosphorylated and total ERK were observed between the study groups 24 h after the OGD procedure (the *p*-values ranged from 0.0994 to above 0.9999) (Figure 5E–H). However, administration of SB at 72 h after OGD increased the level of total ERK (OGD vs. OGD+SB) (*p*-value = 0.0402) (Figure 5F). Givinostat administration did not affect protein levels of total and phosphorylated ERK at this time point (72 h) (the *p*-values ranged from 0.2511 to above 0.9999) (Figure 5G,H).

### 2.4. Effects of HDAC Inhibitors on Selected Signaling Pathways in Microglia After OGD Procedure—ELISA

**Figure 6:**
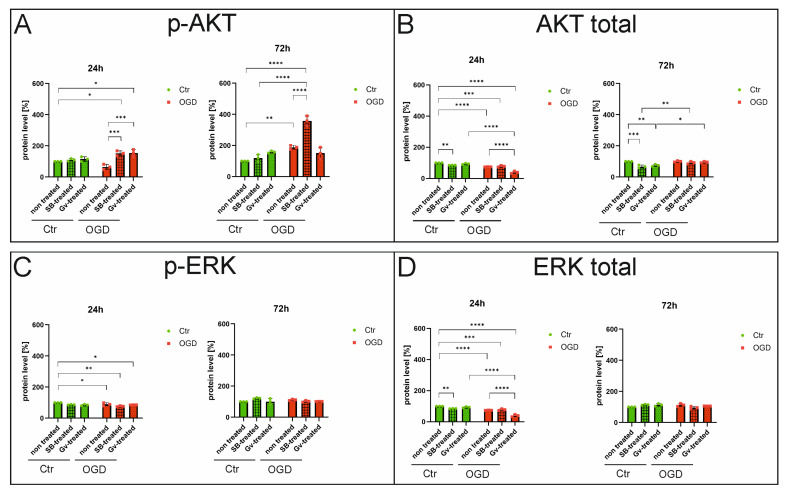
The effect of the histone deacetylase inhibitors on selected signaling pathways after the OGD procedure—ELISA tests. An analysis was performed using dedicated ELISA immunoenzymatic tests on material derived from BV2 microglial cell cultures that underwent the OGD procedure or control cells, in the presence or absence of sodium butyrate (in concentration 1 mM) or Givinostat (in concentration 1 μM). The material was obtained 24 h and 72 h after the OGD procedure. The total protein concentration in the cell homogenates was determined using the Bradford assay (Sigma-Aldrich, St. Louis, MO, USA). Concentrations of selected proteins of the PI3K/AKT and MAPK/ERK signaling pathways in the samples were determined using commercially available immunoenzymatic ELISA kits according to protocols provided by the manufacturers. Absorbance reading at 450 nm was performed on a FluoStar Omega spectrophotometer (BMG Labtech, Ortenberg, Germany). To normalize the results obtained by ELISA, the obtained concentrations of the analyzed proteins were related to the concentration of total protein in the sample. The results for the control samples were taken as 100%, and the data from the corresponding experimental groups were presented as a percentage of the control value. Detailed information about the methodology and ELISA kits used can be found in Section 4.5 in the Materials and Methods Section. (**A**)—analysis results for p-AKT, (**B**)—analysis results for total AKT, (**C**)—analysis results for p-ERK, (**D**)—analysis results for total ERK. Ctr—control, OGD—oxygen-glucose deprivation. Protein concentration results were expressed as a percentage of control SB-treated, control Gv-treated, OGD non-treated, OGD SB-treated, OGD Gv-treated, and bands relative to control non-treated. Color green—controls, color red—OGD. Results are presented as mean values ± standard deviation (SD). Number of biological replicates in each experimental group n = 3. Statistical analysis: two-way ANOVA with Bonferroni post hoc test: **** *p* < 0.001, *** *p* < 0.001, ** *p* < 0.01, * *p* < 0.05.

To verify the results determining the effect of sodium butyrate or Givinostat on selected signaling pathways involved in microglia activation, obtained by Western blot, dedicated ELISA immunoassays were performed on cell lysates of BV2 microglia cultures subjected to the OGD procedure, in the presence of SB (in concentration 1 mM) or Givinostat (in concentration 1 μM) and the absence of these inhibitors, and on control cultures. Material was collected 24 and 72 h after the OGD procedure. Similarly to Western blot, there was a decrease in the phosphorylated form of AKT (p-AKT) 24 h after the OGD procedure compared to control cells (*p*-value = 0.2035). At the same time, administration of both inhibitors, sodium butyrate and Givinostat, following OGD, increased the level of this protein (for SB-treated, *p*-value = 0.0002; for Gv-treated, *p*-value = 0.0001) (Figure 6A). Total AKT protein levels also decreased after the OGD procedure compared to control cultures at the 24 h time point (*p*-value < 0.0001), while SB administration in this case did not cause any statistically significant differences in the levels of this form of protein, compared to cells after the OGD procedure without inhibitor administration (*p*-value > 0.9999). On the other hand, the administration of Givinostat caused a decrease in this protein compared to the OGD-treated group without the addition of inhibitors (*p*-value < 0.0001) (Figure 6B). When cell culture was extended to 72 h, there was a significant increase in the level of p-AKT after OGD (*p*-value = 0.0071), while the administration of sodium butyrate again, as with Western blot, further increased this level (*p*-value < 0.0001). The administration of Givinostat did not cause any statistically significant differences in the levels of this form of protein, compared to cells after the OGD procedure without inhibitor administration (*p*-value = 0.9993) (Figure 6A). At this time point, there were no statistically significant differences in AKT total protein levels after OGD compared to control microglia and SB or Givinostat administration after OGD compared to the non-treated samples (*p*-values > 0.9999) (Figure 6B).

At 24 h after the OGD procedure, there was a slight decrease in the level of the phosphorylated form of ERK protein (p-ERK) compared to control cells (*p*-value = 0.2756). Administration of SB or Givinostat did not influence the level of p-ERK after OGD (OGD+SB/OGD+Gv vs. OGD) (for SB treatment, *p*-value = 0.1099; for Gv-treatment, *p*-value > 0.9999) (Figure 6C). A similar pattern in protein levels was noted for total ERK at this time point (24 h). The OGD procedure caused a decrease in the total form of ERK compared to the control (*p*-value = 0.0001), with no effect of HDACis administration (for SB treatment, *p*-value = 0.2363; for Gv-treatment, *p*-value > 0.9999) (Figure 6D). There were no differences in the levels of p-ERK and ERK total proteins in OGD-treated, SB-treated, and Givinostat-treated cultures after extending the culture time to 72 h (the range of *p*-values was from 0.0854 to above 0.9999) (Figure 6C,D).

## 3. Discussion

In the present study, we investigated the effects of two histone deacetylase inhibitors (HDACi)—sodium butyrate (SB) and Givinostat—on microglial polarization following oxygen-glucose deprivation (OGD), an established in vitro model that mimics hypoxic–ischemic conditions. Our findings demonstrate a divergent impact of these HDACis on the phenotype of microglial cells. Specifically, SB significantly suppressed the mRNA expression of pro-inflammatory markers (IL-1β, TNF-α, and CD86) in BV2 microglial cells after OGD, whereas Givinostat failed to attenuate these inflammatory responses.

Microglia activation and polarization are crucial in post-ischemic development of neuroinflammation and tissue repair following ischemic brain damage. After ischemia, microglia cells begin intensive production of various pro-inflammatory factors—such as cytokines, chemokines, nitric oxide synthase (NOS), and reactive oxygen species (ROS)—which contribute to neuronal dysfunction and ultimately lead to neuronal death [3,28]. Similar results were also obtained in several in vitro studies using primary microglia and the OGD model. Zhang and Zhao demonstrated increased expression of the pro-inflammatory cytokines *TNF-α*, *IL-1β*, and *IL-6* in mouse primary microglial cells 24 h after OGD [29]. Also, our results demonstrated that in vitro hypoxia–ischemia increases the mRNA expression of pro-inflammatory cytokines (*IL-1β*, *TNF-α*) as well as a key activation marker for pro-inflammatory microglia—the surface protein CD86, which is consistent with the findings reported by other authors described above. Some in vivo and in vitro studies have shown that HDAC inhibitors influence microglial activation and polarization following hypoxic–ischemic brain injury [18,30,31]. The results of our present study remain consistent with previous reports showing that SB can induce transition of microglia from an unfavorable M1 to a beneficial M2 phenotype under ischemic conditions, enhancing neurotrophic and anti-inflammatory signaling, and consequently reducing brain injury [21,23,32]. Patnala and coworkers demonstrated that the neuroprotective effect of SB in ischemic mice was correlated with the HDACi—specific immunosuppression of pro-inflammatory mediators (TNF-α and NOS2) and upregulation of the anti-inflammatory gene *IL-10* and activation of the STAT3 signaling pathway in microglia [32]. Moreover, HDAC inhibition by sodium butyrate markedly altered the microglial transcriptome, influencing biological pathways related to neuroinflammation, neuroprotection, and phagocytosis, as well as changed microglial morphology, promoting a shift toward reparative, neurotrophic phenotypes within the ischemic penumbra in the MCAO mice model [33]. Our previous study also indicates that the presence of sodium butyrate increases the number of M2 microglia/macrophages, reduces M1 phenotype cells, and decreases the level of the pro-inflammatory cytokine IL-1β in the ipsilateral (injured) hemisphere in a neonatal rat model of hypoxic–ischemic injury [17,21,34].

Sodium butyrate, a short-chain fatty acid, is a non-specific inhibitor that suppresses both class I and II of HDACs. Butyrate was the first endogenous substance identified to inhibit HDAC and remains the most powerful inhibitor among natural compounds [35]. Sodium butyrate, through HDAC inhibition, leads to increased acetylation of histones, which promotes the transcription of genes associated with the reduction in apoptosis, oxidative stress, and inflammation, and increases the expression of neurotrophic factors (BDNF, NGF), resulting in neuroregeneration and tissue repair following brain ischemia. Moreover, histone deacetylase inhibitors regulate acetylation of a plethora of non-histone proteins such as transcriptional factors (e.g., NFκβ, STAT3, HIF-1α), cytoskeletal proteins (e.g., α-tubulin), and signaling molecules (e.g., HSP90), which suggests that the neuroprotective effects of sodium butyrate may result from their pleiotropic action [36].

In contrast, Givinostat (ITF2357), a HDAC inhibitor containing hydroxamic acid, with strong activity against class I and II HDACs, did not exhibit the anti-inflammatory effects in our experimental model; on the contrary, it increased the mRNA expression levels of IL-1β and TNF-α at both time points examined after OGD. Although there are reports confirming the anti-inflammatory properties of Givinostat in systemic inflammatory diseases and some neurodegenerative models [26,27,37], its ability to modulate microglial activation in the context of ischemic-like injury appears limited. Our previous publication also showed that in a rat model of neonatal HI, Givinostat did not change the level of most investigated chemokines and cytokines induced by HI. The only immunosuppressive effect of Givinostat was associated with the decrease in chemokine MIP-1α [38]. This discrepancy between the anti-inflammatory effect of SB and Givinostat may stem from differences in the selectivity of particular agents or/and downstream targets [26].

The results obtained from flow cytometry analysis did not fully confirm the findings from the qPCR experiments. In these studies, we demonstrated that almost 100% of control BV2 cells expressed the CD86 marker. However, BV2 cells are known to display a partially activated phenotype even under control conditions, likely due to their immortalized nature and the absence of neuron—or astrocyte—derived regulatory signals [39]. Thus, the basal expression of CD86 observed in control BV2 cells likely reflects inherent activation rather than a pro-inflammatory response. Also, after OGD, most of the BV2 cells expressed the CD86 marker without any effect of applied HDAC inhibitors. Similarly, no differences were detected in the proportion of the anti-inflammatory population (CD11b+CD206+) between groups at the first time point after OGD. However, 72 h after the OGD, a decrease in the number of CD11b+CD206+ cells was noted in the OGD group compared to the control. Administration of sodium butyrate after OGD resulted in an increase in the proportion of the anti-inflammatory population in the analyzed samples. Unexpectedly, we also observed some microglia cells express both CD86 and CD206. Similar results were obtained by Zhang et al. [40] in murine models of retinal degeneration. The authors suggested that CD86+CD206+ microglia were involved in phagocytosis rather than the development of inflammation. The double-stained CD86 (+)/CD206 (+) microglia cells were visible in the injured brain tissue after neonatal hypoxia [41]. This phenomenon reflects the emerging concept that microglial activation exists along a spectrum rather than a strict M1/M2 dichotomy [42,43].

In order to further explore the anti-inflammatory mechanism of HDACis, we also studied the effect of SB and Givinostat on ERK and AKT expression and phosphorylation in microglial cells subjected to OGD. Western blot and ELISA results showed that SB increased the phosphorylated level of AKT (p-AKT) in BV2 compared to non-treated controls at both 24 and 72 h after OGD. These results suggest that SB may attenuate microglia-induced inflammation following hypoxic–ischemic injury, probably through activation of the pro-survival AKT pathway. In contrast, the second investigated inhibitor, Givinostat, did not produce a comparable effect. A slight increase in AKT activity/phosphorylation was detected only 24 h post-OGD, as assessed by ELISA.

The serine/threonine kinase AKT plays a pivotal role in regulating microglial physiology and activation. AKT is a key enzyme in the phosphoinositide 3-kinase (PI3K)–AKT signaling pathway, which is activated in microglia downstream of receptors such as CSF-1R, TREM2, CX3CR1, and toll-like receptors (TLRs) [44,45]. Upon ligand engagement, PI3K catalyzes the conversion of phosphatidylinositol 4,5-bisphosphate (PIP_2_) to phosphatidylinositol 3,4,5-trisphosphate (PIP_3_), leading to membrane recruitment and subsequent phosphorylation of AKT. Activated AKT in turn phosphorylates a broad range of substrates, including GSK3β, mTOR, FOXO transcription factors, and NF-κB, thereby orchestrating diverse cellular outcomes such as survival, metabolism, motility, and immune modulation [46]. There are numerous reports indicating that in microglia, AKT activity is tightly linked to phenotypic polarization. Enhanced AKT signaling promotes a neuroprotective, anti-inflammatory M2 phenotype, characterized by increased expression of interleukin-10 (IL-10), IL-1 receptor antagonist (IL-1Ra), and interferon-β (IFN-β). Conversely, suppression of AKT signaling shifts microglia toward a pro-inflammatory M1 phenotype, resulting in elevated production of nitric oxide, TNF-α, IL-1β, IL-6, and other inflammatory mediators [47,48,49]. The effect of HDAC inhibitors on microglial polarization through pathway activation has not yet been clearly defined. Most available data indicate that the use of HDAC inhibitors activates the PI3K/AKT signaling pathways in microglial cells, reduces inflammation, and increases the expression of markers associated with M2 polarization [18,23,50]. Huang and coworkers studied the effect of HDACs inhibition using VPA and TSA on microglial shape, ramification, and polarization, showing that both inhibitors increased AKT phosphorylation levels in primary cultured microglia, which was correlated with induction of M2 polarization [50]. Similarly, another HDAC inhibitor, Scriptaid, enhanced AKT phosphorylation and shifted microglia polarization toward the protective M2 phenotype in LPS-treated primary microglia culture [18]. The authors demonstrated that the protective role of HDAC inhibition in the treatment of white matter injury induced by traumatic brain injury is correlated with the modulation of microglia/macrophage activity by PI3K/AKT. This signaling pathway is also involved in the development of post-ischemic brain injury [51]. Kim and coworkers have shown that the level of phosphorylated AKT was reduced in rats’ brains after ischemia (pMCAO). The treatment with HDAC inhibitors (TSA or SB) restored p-AKT levels to those observed in control animals [23]. Neuroprotective effects of HDAC inhibitors against cerebral ischemia-induced brain damage probably involve multiple mechanisms, including activation of PI3K/AKT signaling and suppression of post-ischemic inflammation.

In contrast to the response of AKT kinase activity, the kinase ERK1/2 (total and phosphorylated form) mostly remained at the control level during the entire course of the current study. Therefore, it does not seem to be involved in phenotype modulation of microglial cells in ischemic conditions. The extracellular signal-regulated kinases 1 and 2 (ERK1/2) are key effectors in the MAPK signaling cascade. Activated (phosphorylated) ERK1/2 translocate into the nucleus, where it engages multiple transcription factors, modulates gene expression, and influences a range of cellular functions, finally inducing repair processes or cell death [52]. It is well-known that ERK1/2 is abnormally expressed in various models of cerebral ischemia. However, the effects of the ERK1/2 signaling and the underlying mechanism vary across different models of ischemia [53,54,55]. In particular, ERK activation in microglia modulates inflammatory signaling, phagocytic activity, and cytokine expression, contributing to both injury propagation and resolution. In rodent models of focal cerebral ischemia, phosphorylation of ERK1/2 is rapidly induced in the ischemic core and peri-infarct regions. Pharmacological inhibition using MEK inhibitors such as U0126 significantly reduces infarct volume and suppresses IL-1β mRNA expression—demonstrating that ERK-dependent activation of Elk-1 transcription factor drives pro-inflammatory cytokine production by microglia/macrophages [56]. Temporal and cell-specific analyses have revealed that microglia are among the earliest responders to ischemic insult, with phosphorylated ERK (p-ERK) localized primarily to Iba1^+^ microglia/macrophages within the first few hours post-injury (<4 h). Notably, treatment with U0126 not only dampens ERK signaling but also significantly reduces microglial activation, cellular proliferation, and glial scar formation, highlighting the role of ERK in modulating the neuroinflammatory response and gliosis [57]. There are reports highlighting the role of ERK in regulating microglial phagocytosis. Following tMCAO, activation of BK potassium channels enhances p-ERK1/2 expression in microglia and promotes clearance of neuronal debris [58]. Finally, emerging studies also implicate ERK in the modulation of microglial polarization states. Activation of ERK can suppress NF-κB nuclear translocation in microglia, thereby inhibiting pro-inflammatory M1 polarization and shifting toward anti-inflammatory M2 phenotypes that support tissue repair [59].

Interestingly, inhibition of histone deacetylases by valproic acid or sodium butyrate does not significantly affect the activation of MAPK signaling pathways, including ERK1/2, p38, or JNK in lipopolysaccharide (LPS)-stimulated primary microglial cultures [19]. This observation suggests that the anti-inflammatory actions of HDAC inhibitors may be mediated primarily through epigenetic reprogramming—such as increased histone acetylation and transcriptional regulation of inflammation-related genes—rather than direct interference with classical MAPK signaling cascades. As mentioned above, ERK1/2 influences microglial activation and polarization in various neuroinflammatory and ischemic conditions, but HDACi-induced shifts from the pro-inflammatory (M1) to the anti-inflammatory (M2) phenotype seem to occur independently of ERK modulation. Instead, HDAC inhibitors appear to exert their effects by enhancing histone acetylation at promoters of anti-inflammatory genes, such as *IL-10*, and repressing transcription of pro-inflammatory mediators through mechanisms involving chromatin remodeling rather than MAPK signaling cascade [32,60].

In summary, our results suggest that not all HDAC inhibitors exert uniform effects on microglial polarization. Sodium butyrate, but not Givinostat, promotes a shift in microglia toward an anti-inflammatory M2 phenotype under ischemic conditions. This effect is associated with suppression of pro-inflammatory gene expression and activation of the PI3K/AKT signaling pathway. SB appears to be a promising candidate for promoting anti-inflammatory microglial phenotypes following injury, whereas the efficacy of Givinostat may be limited or dependent on conditions not replicated in the current model. However, our study has several limitations: (1) the effect of SB on microglial polarization observed in this study is limited to transcriptional changes and does not directly reflect protein secretion or functional activity. (2) Immunofluorescence and morphological analyses would provide valuable complementary insights into microglial phenotypes and should be considered in future studies. (3) Although the effect of SB on shifting microglial polarization from the inflammatory M1 phenotype toward the protective M2 phenotype after OGD was associated with activation of AKT kinase, these associations do not demonstrate causality. The use of specific AKT inhibitors would be necessary to directly confirm that the mechanism of action of SB is mediated through activation of the PI3K/AKT signaling pathway.

## 4. Materials and Methods

### 4.1. Culture of BV2 Microglia Cell Line—In Vitro Model

**Figure 7:**
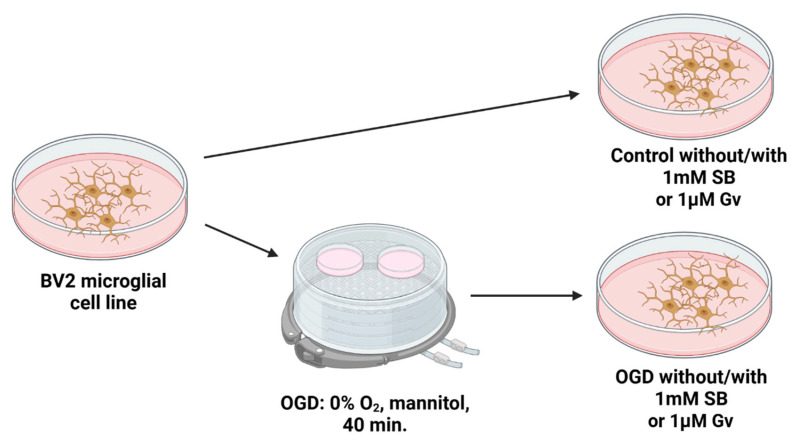
Schematic of the microglia cell line BV2 culture. In further stages, cultures were conducted at 37 °C, in an atmosphere of 5% O_2_, 5% CO_2_ and 95% humidity. Material for analysis was collected at two time points: 24 and 72 h, counting from the day of the OGD procedure, or an analogous time for control cultures.

Microglia cell line BV2 (Elabscience Biotechnology Inc., Wuhan, China, #EP-CL-0493) was cultured in DMEM GlutaMAX High Glucose medium (Dulbecco’s Modified Eagle Medium, Gibco, Waltham, MA, USA, #31966-021) supplemented with 10% fetal bovine serum (FBS, Gibco, #A5256801) and 1% antibiotic (antibiotic-antimycotic solution—AAS, Gibco, #A5955-100ML) according to the manufacturer’s instructions, in 10 cm diameter culture dishes (Nunclon, Roskilde, Denmark), in an incubator under conditions of 5% O_2_, 5% CO_2_, 37 °C. Cells were passaged after they reached 80% confluence. Hypoxic–ischemic injury of cultured cells was induced by eliminating glucose and oxygen from the environment for 40 min (oxygen and glucose deprivation—OGD procedure). For this purpose, the cell cultures were placed in a buffer where glucose was replaced with Ringer’s solution containing 10 mM mannitol (Sigma-Aldrich, St. Louis, MO, USA, #M4125-100G). After the cultures were washed for 10 min with a 95% N_2_/5% CO_2_ gas mixture, the cells were incubated in an anaerobic chamber filled with an oxygen-free gas mixture—a modified method of Fernández-López et al. [61]. After the OGD procedure, the mannitol buffer was replaced with fresh medium or fresh medium supplemented with sodium butyrate (SB; Sigma-Aldrich, #B5887-1G) at a concentration of 1 mM, based on our previously published results [31] or fresh medium supplemented with Givinostat (Gv, Sigma-Aldrich, #SML1772-5MG) at a concentration of 1 μM, based on our previously published results [39]. In control cultures, the medium was replaced with fresh medium at the same time. In addition, some of the control cultures were cultured in a medium supplemented with SB or Gv at the same concentration (Figure 7). Supplementation with inhibitors was followed immediately after the end of OGD—for cultures with an endpoint of 24 h or immediately after the end of OGD and 48 h after OGD (and at the same time for the control groups)—for cultures with an endpoint of 72 h.

The cultures were assigned to 6 experimental groups, according to the following pattern:

(1) control (cultures in which the OGD procedure was not performed) (Ctr);
(2) control treated with sodium butyrate, to evaluate potential negative effects of sodium butyrate administration in control cultures (Ctr+SB);
(3) control treated with Givinostat, to evaluate potential negative effects of Givinostat administration in control cultures (Ctr+Gv);
(4) cultures after the OGD procedure (OGD);
(5) cultures after the OGD procedure treated with sodium butyrate (OGD+SB)
(6) cultures after the OGD procedure treated with Givinostat (OGD+Gv)

### 4.2. Reverse Transcription and Quantitative PCR Analysis

The effect of sodium butyrate and Givinostat on microglia polarization and gene expression of selected pro-inflammatory markers (IL-1β, TNF-α, CD86) and anti-inflammatory factors (IL-4, arginase, CD206) was determined in cultures after OGD procedure and control cultures at different experimental time points. The collected cells were homogenized in liquid nitrogen.

Total RNA was isolated using the Total RNA Mini Kit (A&A Biotechnology, Gdynia, Poland, #AA-031-100), according to the manufacturer’s instructions. The reverse transcription reaction was carried out using High-Capacity RNA-to-cDNA Kit (Applied Biosystems, Foster City, CA, USA, #4387406), according to the manufacturer’s recommendations. The analysis of changes in the mRNA level of genes was carried out using SYBR™ Green PCR Master Mix Reagent (Applied Biosystems, #4385612), 2 μL of cDNA samples, and specifically designed primers (Table 3). The quantitative PCR reactions were performed in the 7500 Fast Real-Time PCR System (Applied Biosystems). The reaction steps were as follows: (1) holding stage: 20 s at 50 °C, 10 min at 95 °C; (2) cycling stage (40×): 15 s at 95 °C, 60 s at 60 °C and 45 s at 72 °C; and (3) melt curve stage: 15 s at 95 °C, 1 min at 60 °C, 30 s at 95 °C, 15 s at 60 °C. Each sample was tested in triplicate during two analysis sessions. The dissociation curve will be plotted to determine the specificity of the amplification. The fluorescence signals of a specific transcript were normalized against those of the reference gene (*β-actin*), and the threshold cycle values (ΔCt) were quantified as fold changes using the 2^−ΔΔCT^ method.

### 4.3. Flow Cytometry

The effect of sodium butyrate and Givinostat on microglia polarization was determined in cultures after the OGD procedure and control cultures at different experimental time points (24 h and 72 h). Flow cytometry analysis was performed to determine the M1 and M2 microglia populations after the OGD procedure and the application of sodium butyrate and Givinostat, and in control cells. For this purpose, antibodies against characteristic markers for both populations were used (Table 4). The cells were labeled CD11b (rat anti-mouse, conjugated with APC, BD Pharmingen™, San Jose, CA, USA), CD86 (rat anti-mouse, conjugated with BV421, BD Pharmingen™) and CD206 (rat anti-mouse, conjugated with PE, BD Pharmingen™) antibodies. To block non-specific binding of Fc receptors, samples were incubated for 15 min at room temperature, in 500 μL total volume, in Hank’s balanced salt solution without calcium and magnesium (HBSS, Gibco, #H6648) with 10% FBS. Antibodies were diluted in 5% FBS in HBSS at a 1:200 concentration. For labeling, samples were incubated for 30 min at room temperature, in a 200 μL total volume. After incubation, the samples were washed three times in 1 mL of 5% FBS solution and transferred into FACS tubes (Falcon™, ThermoFisher Scientific, Waltham, MA, USA).

Flow cytometry was performed using BD FACSCanto™ II flow cytometer (BD Bioscences, San Jose, CA, USA) with excitation lasers of 405 nm, 488 nm and 638 nm, and collecting filters for wave lengths of 450 ± 50 nm (for antibody conjugated with BV421), 585 ± 42 nm (for antibody conjugated with PE), 660 ± 20 nm (for antibody conjugated with APC). The gating strategy was to separate cells based on light scattering on the forward scatter optical detector (FSC) and the side scatter optical detector (SSC) to remove cell aggregates and small debris. The FSC parameter indicates cell size, and the SSC indicates cell granularity. The cell population was determined by excluding the leftmost population (debris and aggregates). Then, the aim was to focus on the gating of single cells based on the parameters of light scattering on the forward detector area (FSC-A) and light scattering on the forward detector height (FSC-H). Single cells were defined as the densest population located along the diagonal of the graph of both parameters. Next, microglia cells were determined by the expression of CD11b. Pro-inflammatory microglial population was defined as CD11b+CD86+, and anti-inflammatory microglial population was defined as CD11b+CD206+. The specification of the used antibodies was verified by adding appropriate isotype antibody controls to the control cells (APC Rat IgG2b, κ Isotype Control, BD Pharmingen™, #553991; BV421 Rat IgG2a, κ Isotype Control, BD Pharmingen™, #562602; PE Rat IgG2a, κ Isotype Control, BD Pharmingen™, #553930).

### 4.4. Biochemical Analysis of Protein Expression Levels by Western Blot Analysis

To evaluate the effects of sodium butyrate on selected signaling pathways involved in microglia activation after hypoxic–ischemic injury, Western blot analysis was performed on the BV2 line of microglia cells 24 and 72 h after the OGD procedure and after treatment with sodium butyrate. For Western blot analysis, the collected cells were homogenized in a RIPA lysis buffer (10 mM Tris-HCl pH 7.5 containing 150 mM NaCl (Sigma-Alrich, #793566-500G), 1% Nonidet P40 (Sigma-Aldrich, #74385), 0.1% SDS (Sigma-Aldrich, #7910-OP), 1% Triton X-100 (Sigma-Aldrich, # X100-1L), PMSF 0.1 mg/mL (Sigma-Aldrich, # P7626-5G)) supplemented with proteinase and a phosphatase inhibitor cocktail (1:100, Sigma Aldrich, #P8340) for 30 min on ice. The lysates were centrifuged at 13,000× *g* for 10 min. at 4 °C, and the supernatants were collected. Total protein concentrations were assessed using a Bio-Rad DCTM protein assay kit (Bio-Rad, #5000111EDU). Lysates were incubated in a water bath with denaturing buffer (Laemmli Sample Buffer, BioRad, #161-0737) at a ratio of 1:1 at 100 °C for 5 min. The denaturing buffer contained an addition of β-mercaptoethanol (BioRad, #161-0710)—1% *v*/*v*, which degrades tertiary and quaternary protein structures. Samples containing 50 µg of protein were separated by SDS–PAGE electrophoresis on a 10% polyacrylamide gel in a buffer composed of 25 mM Tris (Bio-Rad, ##161-0716), 192 mM glycine (IBI Scientific, #IB70194, 0.5% SDS (Sigma-Aldrich, #7910-OP), at a constant voltage of 150 V for approximately 75 min. Each sample was tested at a minimum in duplicate, and each variant in three biological replicates. After electrophoresis, the proteins were transferred from the polyacrylamide gel to nitrocellulose (Amersham™ Protran™ Supported 0.45 μm NC, #10600018) in a buffer containing 25 mM Tris (Bio-Rad, ##161-0716), 192 mM glycine (IBI Scientific, Peosta, IA, USA, #IB70194, 20% methanol (Sigma-Aldrich, # 34885-1L-R), at a constant voltage of 100V for approximately 90 min. The proteins on the nitrocellulose membrane were then subjected to immunochemical analysis. After blocking in 5% non-fat milk solution in TBST (a mixture of buffered Tris saline solution (TBS)—20 mM Tris (Bio-Rad, ##161-0716), 150 mM NaCl (Sigma-Aldrich, #S9888-1KG) and 0.1% Tween 20 (polysorbate 20—nonionic surfactant) (Sigma-Aldrich #P1379-500ML), the membranes were incubated overnight at 4 °C with the appropriate primary antibody. (Table 5). In the next step of Western blot analysis, the membranes were rinsed 3 times in TBST buffer and then incubated for 1 h at room temperature with an anti-rabbit or anti-mouse horseradish peroxidase-conjugated secondary antibody (Sigma-Aldrich) (Table 6). To verify an equal protein loading per line, a mouse monoclonal anti-β-actin (Cell Signaling, 1:1000) was used as an internal control for each Western blot analysis. Immunoblot signals were visualized using an ECL chemiluminescence kit (Amersham™, #RPN2105) by exposure of the membrane to an X-ray Hyperfilm^™^ ECL film (Amersham™, #28906837). A semiquantitative estimation of protein levels detected by immunoblotting was performed utilizing the LKB Utrascan XL v. 2.1 Program GelScan software. The densitometry values were averaged in all groups, and then the densitometry values in the control groups were taken as 100%. The densitometric values are presented in Table S1. The data from the respective experimental groups are presented as percentages of the control value.

### 4.5. Biochemical Analysis of Protein Expression Levels by Platelet Immunoassay-ELISA

To evaluate the effect of sodium butyrate on selected signaling pathways involved in microglia activation after hypoxic–ischemic injury, biochemical analysis of protein expression levels by platelet immunoenzymatic assay (ELISA) was performed on the BV2 line of microglia cells 24 and 72 h after OGD procedure and after sodium butyrate treatment.

At selected time points, cell cultures were washed with PBS, then Cell Extraction Buffer PTR (included) with Protease Inhibitor Cocktail (1:100; Sigma-Aldrich, #P8340) was added in a volume of 400 µL per well and incubated on ice for 20 min. The cells were then collected into tubes and incubated on ice for 15 min. In the next step, the cell lysates were centrifuged for 20 min at 18,000× *g* at 4 °C. The obtained supernatants were placed in new sterile tubes and stored at −80 °C for further analysis. The total protein concentration in the cell homogenates was determined using the Bradford assay (Sigma-Aldrich, #B6916-500ML). Standard solutions of bovine serum albumin (Thermo Scientific™ Pierce™, #PI23210) were used for the standard curve. Absorbance (595 nm) was read on a Fluostar Omega spectrophotometer (BMG Labtech, Ortenberg, Germany). Concentrations of selected proteins of the PI3K/AKT and MAPK/ERK signaling pathways in the samples were determined using commercially available immunoenzymatic ELISA kits (Table 7) according to protocols provided by the manufacturers. Absorbance reading at 450 nm was performed on a Fluostar Omega spectrophotometer (BMG Labtech). To normalize the results obtained by ELISA, the obtained concentrations of the analyzed proteins were related to the concentration of total protein in the sample. The results for the control samples were taken as 100%, and the data from the corresponding experimental groups were presented as a percentage of the control value.

### 4.6. Statistical Analysis

Data analysis was performed using dedicated statistical software (GraphPad Prism 11.0). All results were presented as mean values from individual experimental data, ±standard deviation (SD). Statistical significance analyses were performed on data obtained from at least 3 experiments and 3 technical replicates. Comparisons between groups were performed using two-way analysis of variance (ANOVA) followed by the Bonferroni post hoc test for multiple comparisons. The data were considered statistically significant at *p*-value < 0.05. The data is available in Table S2.

## Figures and Tables

**Table 1:** List of the percentage contribution of individual populations in the analyzed samples 24 h after OGD. CD11b+CD86+ cells were defined as pro-inflammatory phenotype (red), CD11b+CD206+ cells as anti-inflammatory (green), and CD86+CD206+ cells as “transitional” microglia (yellow). Detailed information about the methodology and antibodies used can be found in Section 4.3 in the Materials and Methods Section. Ctr—control cells, Ctr+SB—control cells treated with sodium butyrate, Ctr+Gv—control cells treated with Givinostat, OGD—cells after the OGD procedure, OGD+SB—cells after the OGD procedure treated with sodium butyrate, OGD+Gv—cells after the OGD procedure treated with Givinostat. Number of biological replicates in each experimental group n_1_ = 4. Number of events analyzed in each experimental group n_2_ = 30,000. Results are presented as mean values ± standard deviation (SD).

	Ctr	Ctr+SB	Ctr+Gv	OGD	OGD+SB	OGD+Gv
**% of Singlets**	82.875 ± 21.591	81.2 ± 22.190	86.775 ± 15.691	84.7 ± 18.131	82.3 ± 20.446	88.275 ± 12.053
**% from total events**	59.5 ± 15.44	61.7 ± 17.1401	61.08 ± 13.001	67.65 ± 14.884	63.48 ± 16.026	64.80 ± 11.678
	Ctr	Ctr+SB	Ctr+Gv	OGD	OGD+SB	OGD+Gv
	% from singlets					
**CD11b+CD86−**	0.025 ± 0.043	0 ± 0	0 ± 0	0 ± 0	0 ± 0	0 ± 0
**CD11b+CD206−**	96.475 ± 2.028	95.9 ± 1.7161	95.475 ± 1.780	95.275 ± 1.366	96.225 ± 1.814	95.825 ± 1.457
**CD11b+CD86+**	99.4 ± 0.324	99.2 ± 0.561	98.975 ± 1.203	96.45 ± 3.545	99.525 ± 0.303	98.35 ± 0.921
**CD11b+CD206+**	3.05 ± 2.112	3.8 ± 1.771	3.25 ± 1.787	3.075 ± 1.7302	3.475 ± 1.931	2.775 ± 1.812
**CD86+CD206+**	2.4 ± 1.987	2.775 ± 1.871	2.25 ± 1.05	2.45 ± 1.847	2.725 ± 1.865	2.075 ± 1.923
**CD86+CD206−**	97.45 ± 1.936	97.15 ± 1.890	97.625 ± 0.942	97.45 ± 1.792	97.2 ± 1.893	97.825 ± 1.862
**CD206+CD86−**	0 ± 0	0 ± 0	0 ± 0	0 ± 0	0 ± 0	0 ± 0
**CD11b−CD86+**	0.525 ± 0.349	0.5 ± 0.520	0.875 ± 1.059	3.45 ± 3.545	0.375 ± 0.303	1.55 ± 0.820
**CD11b−CD206+**	0.025 ± 0.043	0 ± 0	0 ± 0	0 ± 0	0 ± 0	0 ± 0

**Table 2:** List of the percentage contribution of individual populations in the analyzed samples 72 h after OGD. CD11b+CD86+ cells were defined as pro-inflammatory phenotype (red), CD11b+CD206+ cells as anti-inflammatory (green), and CD86+CD206+ cells as “transitional” microglia (yellow). Detailed information about the methodology and antibodies used can be found in Section 4.3 in the Materials and Methods Section. Ctr—control cells, Ctr+SB—control cells treated with sodium butyrate, Ctr+Gv—control cells treated with Givinostat, OGD—cells after the OGD procedure, OGD+SB—cells after the OGD procedure treated with sodium butyrate, OGD+Gv—cells after the OGD procedure treated with Givinostat. Number of biological replicates in each experimental group n_1_ = 4. Number of events analyzed in each experimental group n_2_ = 30,000. Results are presented as mean values ± standard deviation (SD).

	Ctr	Ctr+SB	Ctr+Gv	OGD	OGD+SB	OGD+Gv
**% of Singlets**	92.0 ± 4.003	91.88 ± 0.020	92.6 ± 3.423	92.875 ± 3.642	91.375 ± 4.080	93.2 ± 3.929
**% from total events**	74.1 ± 9.881	59.025 ± 24.533	73.45 ± 10.74	69.45 ± 16.31	70.55 ± 27.12	52.81 ± 31.96
	Ctr	Ctr+SB	Ctr+Gv	OGD	OGD+SB	OGD+Gv
	% from singlets					
**CD11b+CD86−**	0.1 ± 0.071	0.125 ± 0164	0.025 ± 0.0433	0.075 ± 0.083	0.075 ± 0.130	0.05 ± 0.05
**CD11b+CD206−**	88.95 ± 6.500	82.75 ± 9.500	90.125 ± 8.110	93.875 ± 2.249	76.25 ± 12.691	82.2 ± 19.973
**CD11b+CD86+**	96.95 ± 3.725	97.55 ± 3.677	97.575 ± 3.269	98.9 ± 1.332	91.7 ± 12.746	88.275 ± 18.389
**CD11b+CD206+**	8.475 ± 4.402	15.375 ± 10.883	7.825 ± 6.208	5.275 ± 1.295	16.375 ± 3.628	7.15 ± 5.1
**CD86+CD206+**	7.9 ± 4.294	14.075 ± 9.944	7.475 ± 6.085	4.775 ± 1.295	16.725 ± 5.007	8.35 ± 6.881
**CD86+CD206−**	91.625 ± 4.492	85.55 ± 9.622	92.3 ± 6.313	94.9 ± 1.608	82.675 ± 5.55	90.825 ± 7.768
**CD206+CD86−**	0 ± 0	0 ± 0	0 ± 0	0 ± 0	0 ± 0	0.075 ± 0.130
**CD11b-CD86+**	2.6 ± 3.348	2.1 ± 3.407	2.15 ± 3.166	0.75 ± 0.955	7.625 ± 12.356	10.875 ± 17.367
**CD11b-CD206+**	0.3 ± 0.354	0.125 ± 0.164	0.325 ± 0.455	0.025 ± 0.043	1.825 ± 2.935	2.075 ± 3.423

**Table 3:** List of designed primers used in quantitative real-time PCR analysis.

Gene Starter	Sequence (5′-3′)	Melting Temperature (T_M_) [°C]	GC Value [%]
IL-1β forward	CCACCTTTTGACAGTGATGA	49.7	45.0
IL-1β reverse	GAGATTTGAAGCTGGATGCT	49.7	45.0
TNF-α forward	CCCTCCAGAAAAGACACCATG	54.4	52.4
TNF-α reverse	GCCACAAGCAGGAATGAGAAG	54.4	52.4
CD86 forward	TCTCCACGGAAACAGCATCT	51.8	50.0
CD86 reverse	CTTACGGAAGCACCCATGAT	51.8	50.0
IL-4 forward	ATCGGCATTTTGAACGAGGTCACA	55.7	45.8
IL-4 reverse	CGAAGCACCTTGGAAGCCCTA	56.3	57.1
Arg1 forward	AGGAAAGCTGGTCTGCTGGAA	54.4	52.4
Arg1 reverse	GATGCTTCCAACTGCCAGAC	54.4	52.4
CD206 forward	CTCAACCCAAGGGCTCTTCTAA	54.8	50.0
CD206 reverse	AGGTGGCCTCTTGAGGTATGTG	56.7	54.5
β-actin forward	CTGAGAGGGAAACGTGCGT	53.8	55.0
β-actin reverse	CCACAGGATTCCATACCCAAGA	54.8	50.0

**Table 4:** List of antibodies used in flow cytometry.

Marker	Antibody	Concentration	Manufacturer	Catalog Number
CD11b	rat anti-mouse, conjugated with APC	1:200 in 5% FBS in HBSS	BD Pharmingen™	553312
CD86	rat anti-mouse, conjugated with BV421	1:200 in 5% FBS in HBSS	BD Pharmingen™	564198
CD206	rat anti-mouse, conjugated with PE	1:200 in 5% FBS in HBSS	BD Pharmingen™	568273

**Table 5:** List of primary antibodies used in Western blot.

Protein	Primary Antibody	Concentration	Manufacturer	Catalog Number
phospho-AKT (Ser473)	rabbit polyclonal antibody IgG anti-p-AKT	1:1000 in TBST	Cell Signaling (Danvers, MA, USA)	9271
AKT	rabbit polyclonal antibody IgG anti-AKT	1:1000 in TBST	Cell Signaling	9272
p-ERK 1/2 (Thr202/Tyr204)	rabbit polyclonal antibody IgG anti-p-ERK	1:1000 in TBST	Affinity Bioscences (Cincinnati, OH, USA)	AF1015
p44/42 MAPK (ERK 1/2)	rabbit polyclonal antibody IgG anti-ERK	1:1000 in TBST	Cell Signaling	9102
β-actin	mouse monoclonal antibody IgG2b anti-β-actin	1:1000 in TBST	Cell Signaling	3700

**Table 6:** List of secondary antibodies used in Western blot.

Antibody	Concentration	Manufacturer	Catalog Number
goat anti-mouse IgG (Fab specific)	1:4000 in 2.5% non-fat milk in TBST	Sigma-Aldrich	12-349
goat anti-rabbit (whole molecule)	1:8000 in 2.5% non-fat milk in TBST	Sigma-Aldrich	9169

**Table 7:** List of used commercially available immunoenzymatic ELISA kits.

ELISA Kit Name	Manufacturer	Catalog Number
AKT 1/2/3 pS473 + AKT 1/2/3 Total ELISA Kit	abcam	ab253299
ERK 1/2 (pT202/Y204 + Total) ELISA Kit	abcam	ab176660

## Data Availability

The data sets used and/or analyzed during the current study are available from the corresponding author on reasonable request.

## Supplementary Materials

The following supporting information can be downloaded at https://www.mdpi.com/article/10.3390/ijms27021114/s1.

## Abbreviations

The following abbreviations are used in this manuscript:

AAS	antibiotic-antimycotic solution
AKT	protein kinase B
ANOVA	analysis of variance
Arg-1	arginase 1
APC	allophycocyanin
BV2	murine microglia cell line
BV421	brilliant violet 421
BBB	blood–brain barrier
BDNF	brain-derived neurotrophic factor
BK	big potassium
CD11b	cluster of differentiation molecule 11b
CD206	cluster of differentiation 206
CD86	cluster of differentiation 86
CNS	central nervous system
COX-2	cyclooxygenase-2
CSF-1R	colony-stimulating factor 1 receptor
CX3CR1	CX3C motif chemokine receptor 1
DMEM	Dulbecco’s Modified Eagle Medium
ECL	electrochemiluminescence
ELISA	enzyme-linked immunosorbent assay
Elk 1	ETS like-1
ERK	extracellular signal-regulated kinase
FACS	fluorescence-activated cell sorting
FBS	fetal bovine serum
FOXO	forkhead box class O
FSC	forward scatter optical detector
FSC-A	forward detector area
FSC-H	forward detector height
GDNF	glial cell line-derived neurotrophic factor
GSK3β	glycogen synthase kinase-3 beta
HATs	histone acetylases
HBSS	Hanks’ Balanced Salt Solution
HDAC	histone deacetylase
HDACis	histone deacetylase inhibitors
HI	hypoxia-ischemia
HIF-1α	hypoxia-inducible factor 1
HSP90	heat shock protein 90
Iba1	ionized calcium-binding adapter molecule 1
IFN-β	interferon beta
IL-1β	interleukin-1 beta, pro-inflammatory
IL-2	interleukin-2, pro-inflammatory
IL-4	interleukin-4, anti-inflammatory
IL-6	interleukin-6, pro-inflammatory
IL-10	interleukin-10, anti-inflammatory
IL-12	interleukin-12, pro-inflammatory
IL-18	interleukin-18, pro-inflammatory
IL-23	interleukin-23, pro-inflammatory
IL-1Ra	interleukin 1 receptor antagonist
ITF 2357	Givinostat
JNK	c-Jun N-terminal kinases
LPS	lipopolysaccharide
MAPK	mitogen-activated protein kinase
MCAO	middle cerebral artery occlusion
MIP-1α	macrophage Inflammatory proteins 1-alpha
MMPs	metalloproteinases
mRNA	messenger ribonucleic acid
mTOR	mammalian target of rapamycin
M1	pro-inflammatory microglia phenotype
M2	anti-inflammatory microglia phenotype
NFκβ	nuclear factor kappa-light-chain-enhancer of activated B cells
NGF	nerve growth factor
NOS	nitric oxide synthase
OD	optical density
OGD	oxygen-glucose deprivation
p-AKT	phosphorylated protein kinase B
PBS	phosphate-buffered saline
PE	phosphatidylethanolamine
p-ERK	phosphorylated extracellular signal-regulated kinase
PIP_2_	phosphatidylinositol 4,5-bisphosphate
PIP_3_	phosphatidylinositol 3,4,5-trisphosphate
PI3K	phosphatidylinositol 3-kinases
pMCAO	permanent middle cerebral artery occlusion
PTEN	phosphatase and tensin homolog
qPCR	quantitative polymerase chain reaction
RIPA	radioimmunoprecipitation assay buffer
ROS	reactive oxygen species
SB	sodium butyrate
SD	standard deviation
SDS	sodium dodecyl sulfate
SDS-PAGE	sodium dodecyl sulfate polyacrylamide gel electrophoresis
SSC	scatter optical detector
STAT3	signal transducer and activator of transcription 3
TBS	tris buffered saline
TBS-T	tris buffered saline Tween
TGF-β	transforming growth factor- beta
TLR	tool-like receptor
tMCAO	transient middle cerebral artery occlusion
TNF-α	tumor necrosis factor alpha, pro-inflammatory
TREM2	triggering receptor expressed on myeloid cells 2
TSA	trichostatin A
WB	Western blot
VPA	valproic acid
