# Supplementary material for: The Diverse Effect of HDAC Inhibitors: Sodium Butyrate and Givinostat on Microglia Polarization After Hypoxia-Ischemia In Vitro
Source: Int J Mol Sci. 2026 Jan 22;27(2):1114. doi: 10.3390/ijms27021114 (PMC12842125; doi:10.3390/ijms27021114)
Supplement: Supplementary file 1 [file ijms-27-01114-s001.zip › ijms-4023957-supplementary/Table S2.pdf]

Table S2. Results of statistical analysis

1. qPCR analysis

IL-1β

24 h

Bonferroni's multiple comparisons test	Mean Diff.	95.00% CI of diff.	Below threshold?	Summary	Adjusted P Value
non-treated:OGD vs. non-treated:Ctr	32.34	17.66 to 47.03	Yes	****	<0.0001
SB-treated:Ctr vs. non-treated:Ctr	4.479	-10.21 to 19.17	No	ns	>0.9999
SB-treated:OGD vs. non-treated:Ctr	3.174	-11.51 to 17.86	No	ns	>0.9999
Gv-treated:Ctr vs. non-treated:Ctr	0.03424	-14.65 to 14.72	No	ns	>0.9999
Gv-treated:OGD vs. non-treated:Ctr	75.37	60.68 to 90.05	Yes	****	<0.0001
SB-treated:Ctr vs. non-treated:OGD	-27.87	-42.55 to -13.18	Yes	***	0.0002
SB-treated:OGD vs. non-treated:OGD	-29.17	-43.86 to -14.48	Yes	***	0.0002
Gv-treated:Ctr vs. non-treated:OGD	-32.31	-47.00 to -17.62	Yes	****	<0.0001
Gv-treated:OGD vs. non-treated:OGD	43.02	28.33 to 57.71	Yes	****	<0.0001
SB-treated:OGD vs. SB-treated:Ctr	-1.305	-15.99 to 13.38	No	ns	>0.9999
Gv-treated:Ctr vs. SB-treated:Ctr	-4.445	-19.13 to 10.24	No	ns	>0.9999
Gv-treated:OGD vs. SB-treated:Ctr	70.89	56.20 to 85.57	Yes	****	<0.0001
Gv-treated:Ctr vs. SB-treated:OGD	-3.14	-17.83 to 11.55	No	ns	>0.9999
Gv-treated:OGD vs. SB-treated:OGD	72.19	57.50 to 86.88	Yes	****	<0.0001
Gv-treated:OGD vs. Gv-treated:Ctr	75.33	60.64 to 90.02	Yes	****	<0.0001

72 h

Bonferroni's multiple comparisons test	Mean Diff.	95.00% CI of diff.	Below threshold?	Summary	Adjusted P Value
non-treated:OGD vs. non-treated:Ctr	43.71	32.50 to 54.91	Yes	****	<0.0001
SB-treated:Ctr vs. non-treated:Ctr	59.04	47.84 to 70.25	Yes	****	<0.0001
SB-treated:OGD vs. non-	29.27	18.06 to	Yes	****	<0.0001

treated:Ctr		40.47			
Gv-treated:Ctr vs. non-treated:Ctr	-0.3814	-11.59 to 10.82	No	ns	>0.9999
Gv-treated:OGD vs. non-treated:Ctr	166.5	155.3 to 177.7	Yes	****	<0.0001
SB-treated:Ctr vs. non-treated:OGD	15.33	4.129 to 26.54	Yes	**	0.0047
SB-treated:OGD vs. non-treated:OGD	-14.44	-25.64 to -3.234	Yes	**	0.0077
Gv-treated:Ctr vs. non-treated:OGD	-44.09	-55.29 to -32.88	Yes	****	<0.0001
Gv-treated:OGD vs. non-treated:OGD	122.8	111.5 to 134.0	Yes	****	<0.0001
SB-treated:OGD vs. SB-treated:Ctr	-29.77	-40.98 to -18.57	Yes	****	<0.0001
Gv-treated:Ctr vs. SB-treated:Ctr	-59.42	-70.63 to -48.22	Yes	****	<0.0001
Gv-treated:OGD vs. SB-treated:Ctr	107.4	96.21 to 118.6	Yes	****	<0.0001
Gv-treated:Ctr vs. SB-treated:OGD	-29.65	-40.85 to -18.45	Yes	****	<0.0001
Gv-treated:OGD vs. SB-treated:OGD	137.2	126.0 to 148.4	Yes	****	<0.0001
Gv-treated:OGD vs. Gv-treated:Ctr	166.8	155.6 to 178.0	Yes	****	<0.0001

TNFα

<u>24 h</u>

Bonferroni's multiple comparisons test	Mean Diff.	95.00% CI of diff.	Below threshold?	Summary	Adjusted P Value
non-treated:OGD vs. non-treated:Ctr	12.02	9.582 to 14.47	Yes	****	<0.0001
SB-treated:Ctr vs. non-treated:Ctr	3.951	1.509 to 6.393	Yes	**	0.0011
SB-treated:OGD vs. non-treated:Ctr	4.745	2.302 to 7.187	Yes	***	0.0002
Gv-treated:Ctr vs. non-treated:Ctr	11.67	9.223 to 14.11	Yes	****	<0.0001
Gv-treated:OGD vs. non-treated:Ctr	15.86	13.42 to 18.30	Yes	****	<0.0001
SB-treated:Ctr vs. non-treated:OGD	-8.074	-10.52 to -5.631	Yes	****	<0.0001
SB-treated:OGD vs. non-treated:OGD	-7.28	-9.723 to -4.838	Yes	****	<0.0001
Gv-treated:Ctr vs. non-treated:OGD	-0.3596	-2.802 to 2.083	No	ns	>0.9999
Gv-treated:OGD vs. non-treated:OGD	3.835	1.392 to 6.277	Yes	**	0.0014

SB-treated:OGD vs. SB-treated:Ctr	0.7936	-1.649 to 3.236	No	ns	>0.9999
Gv-treated:Ctr vs. SB-treated:Ctr	7.714	5.272 to 10.16	Yes	****	<0.0001
Gv-treated:OGD vs. SB-treated:Ctr	11.91	9.466 to 14.35	Yes	****	<0.0001
Gv-treated:Ctr vs. SB-treated:OGD	6.921	4.478 to 9.363	Yes	****	<0.0001
Gv-treated:OGD vs. SB-treated:OGD	11.11	8.672 to 13.56	Yes	****	<0.0001
Gv-treated:OGD vs. Gv-treated:Ctr	4.194	1.752 to 6.637	Yes	***	0.0006

72 h

Bonferroni's multiple comparisons test	Mean Diff.	95.00% CI of diff.	Below threshold ?	Summary	Adjusted P Value
non-treated:OGD vs. non-treated:Ctr	0.3647	-0.3395 to 1.069	No	ns	>0.9999
SB-treated:Ctr vs. non-treated:Ctr	0.5439	-0.1603 to 1.248	No	ns	0.2326
SB-treated:OGD vs. non-treated:Ctr	-0.1254	-0.8296 to 0.5788	No	ns	>0.9999
Gv-treated:Ctr vs. non-treated:Ctr	-0.4208	-1.125 to 0.2834	No	ns	0.7481
Gv-treated:OGD vs. non-treated:Ctr	1.851	1.146 to 2.555	Yes	****	<0.0001
SB-treated:Ctr vs. non-treated:OGD	0.1792	-0.5249 to 0.8834	No	ns	>0.9999
SB-treated:OGD vs. non-treated:OGD	-0.4901	-1.194 to 0.2141	No	ns	0.3895
Gv-treated:Ctr vs. non-treated:OGD	-0.7854	-1.490 to -0.08124	Yes	*	0.0233
Gv-treated:OGD vs. non-treated:OGD	1.486	0.7817 to 2.190	Yes	****	<0.0001
SB-treated:OGD vs. SB-treated:Ctr	-0.6693	-1.373 to 0.03487	No	ns	0.0697
Gv-treated:Ctr vs. SB-treated:Ctr	-0.9647	-1.669 to -0.2605	Yes	**	0.0047
Gv-treated:OGD vs. SB-treated:Ctr	1.307	0.6024 to 2.011	Yes	***	0.0003
Gv-treated:Ctr vs. SB-treated:OGD	-0.2954	-0.9995 to 0.4088	No	ns	>0.9999
Gv-treated:OGD vs. SB-treated:OGD	1.976	1.272 to 2.680	Yes	****	<0.0001
Gv-treated:OGD vs. Gv-treated:Ctr	2.271	1.567 to 2.975	Yes	****	<0.0001

IL-4

24 h

Bonferroni's multiple comparisons test	Mean Diff.	95.00% CI of diff.	Below threshold?	Summary	Adjusted P Value
non-treated:OGD vs. non-treated:Ctr	-0.4313	-2.307 to 1.444	No	ns	>0.9999
SB-treated:Ctr vs. non-treated:Ctr	7.133	5.257 to 9.008	Yes	****	<0.0001
SB-treated:OGD vs. non-treated:Ctr	1.923	0.04771 to 3.799	Yes	*	0.0422
Gv-treated:Ctr vs. non-treated:Ctr	-0.662	-2.538 to 1.214	No	ns	>0.9999
Gv-treated:OGD vs. non-treated:Ctr	1.229	-0.6470 to 3.104	No	ns	0.5118
SB-treated:Ctr vs. non-treated:OGD	7.564	5.688 to 9.440	Yes	****	<0.0001
SB-treated:OGD vs. non-treated:OGD	2.355	0.4790 to 4.230	Yes	**	0.0095
Gv-treated:Ctr vs. non-treated:OGD	-0.2307	-2.106 to 1.645	No	ns	>0.9999
Gv-treated:OGD vs. non-treated:OGD	1.66	-0.2157 to 3.536	No	ns	0.1084
SB-treated:OGD vs. SB-treated:Ctr	-5.209	-7.085 to -3.334	Yes	****	<0.0001
Gv-treated:Ctr vs. SB-treated:Ctr	-7.795	-9.670 to -5.919	Yes	****	<0.0001
Gv-treated:OGD vs. SB-treated:Ctr	-5.904	-7.780 to -4.028	Yes	****	<0.0001
Gv-treated:Ctr vs. SB-treated:OGD	-2.585	-4.461 to -0.7097	Yes	**	0.0044
Gv-treated:OGD vs. SB-treated:OGD	-0.6947	-2.570 to 1.181	No	ns	>0.9999
Gv-treated:OGD vs. Gv-treated:Ctr	1.891	0.01496 to 3.766	Yes	*	0.0474

72 h

Bonferroni's multiple comparisons test	Mean Diff.	95.00% CI of diff.	Below threshold?	Summary	Adjusted P Value
non-treated:OGD vs. non-treated:Ctr	-0.5478	-0.8997 to -0.1960	Yes	**	0.0015
SB-treated:Ctr vs. non-treated:Ctr	-0.6715	-1.023 to -0.3197	Yes	***	0.0002
SB-treated:OGD vs. non-treated:Ctr	-0.2233	-0.5751 to 0.1285	No	ns	0.586
Gv-treated:Ctr vs. non-treated:Ctr	-0.9176	-1.269 to -0.5658	Yes	****	<0.0001
Gv-treated:OGD vs. non-	-0.8044	-1.156 to -	Yes	****	<0.0001

treated:Ctr		0.4526			
SB-treated:Ctr vs. non-treated:OGD	-0.1237	-0.4755 to 0.2281	No	ns	>0.9999
SB-treated:OGD vs. non-treated:OGD	0.3245	-0.02729 to 0.6764	No	ns	0.0842
Gv-treated:Ctr vs. non-treated:OGD	-0.3698	-0.7216 to -0.01795	Yes	*	0.0356
Gv-treated:OGD vs. non-treated:OGD	-0.2566	-0.6084 to 0.09523	No	ns	0.3112
SB-treated:OGD vs. SB-treated:Ctr	0.4482	0.09640 to 0.8001	Yes	**	0.0084
Gv-treated:Ctr vs. SB-treated:Ctr	-0.2461	-0.5979 to 0.1057	No	ns	0.3804
Gv-treated:OGD vs. SB-treated:Ctr	-0.1329	-0.4847 to 0.2189	No	ns	>0.9999
Gv-treated:Ctr vs. SB-treated:OGD	-0.6943	-1.046 to -0.3425	Yes	***	0.0002
Gv-treated:OGD vs. SB-treated:OGD	-0.5811	-0.9330 to -0.2293	Yes	***	0.0009
Gv-treated:OGD vs. Gv-treated:Ctr	0.1132	-0.2386 to 0.4650	No	ns	>0.9999

Arginase-1

<u>24 h</u>

Bonferroni's multiple comparisons test	Mean Diff.	95.00% CI of diff.	Below threshold?	Summary	Adjusted P Value
non-treated:OGD vs. non-treated:Ctr	-0.3557	-5.394 to 4.683	No	ns	>0.9999
SB-treated:Ctr vs. non-treated:Ctr	2.846	-2.193 to 7.884	No	ns	0.9251
SB-treated:OGD vs. non-treated:Ctr	4.61	-0.4281 to 9.649	No	ns	0.0885
Gv-treated:Ctr vs. non-treated:Ctr	4.643	-0.3951 to 9.682	No	ns	0.0847
Gv-treated:OGD vs. non-treated:Ctr	4.306	-0.7325 to 9.344	No	ns	0.1332
SB-treated:Ctr vs. non-treated:OGD	3.201	-1.837 to 8.240	No	ns	0.5831
SB-treated:OGD vs. non-treated:OGD	4.966	-0.07245 to 10.00	No	ns	0.055
Gv-treated:Ctr vs. non-treated:OGD	4.999	-0.03940 to 10.04	No	ns	0.0527
Gv-treated:OGD vs. non-treated:OGD	4.662	-0.3768 to 9.700	No	ns	0.0826
SB-treated:OGD vs. SB-treated:Ctr	1.765	-3.274 to 6.803	No	ns	>0.9999
Gv-treated:Ctr vs. SB-treated:Ctr	1.798	-3.241 to 6.836	No	ns	>0.9999
Gv-treated:OGD vs. SB-treated:Ctr	1.46	-3.578 to 6.499	No	ns	>0.9999
Gv-treated:Ctr vs. SB-treated:OGD	0.03305	-5.005 to 5.071	No	ns	>0.9999
Gv-treated:OGD vs. SB-	-0.3043	-5.343 to 4.734	No	ns	>0.9999

treated:OGD					
Gv-treated:OGD vs. Gv-treated:Ctr	-0.3374	-5.376 to 4.701	No	ns	>0.9999

72 h

Bonferroni's multiple comparisons test	Mean Diff.	95.00% CI of diff.	Below threshold ?	Summary	Adjusted P Value
non-treated:OGD vs. non-treated:Ctr	-0.7368	-0.8307 to -0.6429	Yes	****	<0.0001
SB-treated:Ctr vs. non-treated:Ctr	-0.8284	-0.9223 to -0.7345	Yes	****	<0.0001
SB-treated:OGD vs. non-treated:Ctr	-0.8577	-0.9516 to -0.7638	Yes	****	<0.0001
Gv-treated:Ctr vs. non-treated:Ctr	-0.8403	-0.9342 to -0.7464	Yes	****	<0.0001
Gv-treated:OGD vs. non-treated:Ctr	-0.8899	-0.9838 to -0.7960	Yes	****	<0.0001
SB-treated:Ctr vs. non-treated:OGD	-0.09162	-0.1855 to 0.002279	No	ns	0.0588
SB-treated:OGD vs. non-treated:OGD	-0.1208	-0.2147 to -0.02694	Yes	**	0.0078
Gv-treated:Ctr vs. non-treated:OGD	-0.1035	-0.1973 to -0.009551	Yes	*	0.0255
Gv-treated:OGD vs. non-treated:OGD	-0.1531	-0.2470 to -0.05920	Yes	**	0.001
SB-treated:OGD vs. SB-treated:Ctr	-0.02922	-0.1231 to 0.06468	No	ns	>0.9999
Gv-treated:Ctr vs. SB-treated:Ctr	-0.01183	-0.1057 to 0.08207	No	ns	>0.9999
Gv-treated:OGD vs. SB-treated:Ctr	-0.06148	-0.1554 to 0.03242	No	ns	0.5129
Gv-treated:Ctr vs. SB-treated:OGD	0.01739	-0.07651 to 0.1113	No	ns	>0.9999
Gv-treated:OGD vs. SB-treated:OGD	-0.03226	-0.1262 to 0.06164	No	ns	>0.9999
Gv-treated:OGD vs. Gv-treated:Ctr	-0.04965	-0.1435 to 0.04425	No	ns	>0.9999

CD86

24 h

Bonferroni's multiple comparisons test	Mean Diff.	95.00% CI of diff.	Below threshold?	Summary	Adjusted P Value
non-treated:OGD vs. non-treated:Ctr	7.331	5.523 to 9.139	Yes	****	<0.0001
SB-treated:Ctr vs. non-treated:Ctr	1.816	0.008407 to	Yes	*	0.0485

		3.624			
SB-treated:OGD vs. non-treated:Ctr	0.1543	-1.654 to 1.962	No	ns	>0.9999
Gv-treated:Ctr vs. non-treated:Ctr	-0.3114	-2.119 to 1.497	No	ns	>0.9999
Gv-treated:OGD vs. non-treated:Ctr	5.402	3.594 to 7.210	Yes	****	<0.0001
SB-treated:Ctr vs. non-treated:OGD	-5.514	-7.322 to -3.706	Yes	****	<0.0001
SB-treated:OGD vs. non-treated:OGD	-7.177	-8.985 to -5.369	Yes	****	<0.0001
Gv-treated:Ctr vs. non-treated:OGD	-7.642	-9.450 to -5.834	Yes	****	<0.0001
Gv-treated:OGD vs. non-treated:OGD	-1.929	-3.737 to -0.1209	Yes	*	0.0321
SB-treated:OGD vs. SB-treated:Ctr	-1.662	-3.470 to 0.1459	No	ns	0.086
Gv-treated:Ctr vs. SB-treated:Ctr	-2.128	-3.936 to -0.3198	Yes	*	0.0156
Gv-treated:OGD vs. SB-treated:Ctr	3.586	1.778 to 5.394	Yes	***	0.0002
Gv-treated:Ctr vs. SB-treated:OGD	-0.4657	-2.274 to 1.342	No	ns	>0.9999
Gv-treated:OGD vs. SB-treated:OGD	5.248	3.440 to 7.056	Yes	****	<0.0001
Gv-treated:OGD vs. Gv-treated:Ctr	5.713	3.905 to 7.521	Yes	****	<0.0001

72 h

Bonferroni's multiple comparisons test	Mean Diff.	95.00% CI of diff.	Below threshold?	Summary	Adjusted P Value
non-treated:OGD vs. non-treated:Ctr	-0.4522	-0.6948 to -0.2095	Yes	***	0.0003
SB-treated:Ctr vs. non-treated:Ctr	-0.3245	-0.5671 to -0.08183	Yes	**	0.0057
SB-treated:OGD vs. non-treated:Ctr	-0.7334	-0.9760 to -0.4908	Yes	****	<0.0001
Gv-treated:Ctr vs. non-treated:Ctr	-0.442	-0.6846 to -0.1993	Yes	***	0.0004
Gv-treated:OGD vs. non-treated:Ctr	-0.5137	-0.7563 to -0.2710	Yes	****	<0.0001
SB-treated:Ctr vs. non-treated:OGD	0.1277	-0.1149 to 0.3704	No	ns	>0.9999
SB-treated:OGD vs. non-treated:OGD	-0.2812	-0.5238 to -0.03858	Yes	*	0.0175
Gv-treated:Ctr vs. non-treated:OGD	0.0102	-0.2324 to 0.2528	No	ns	>0.9999
Gv-treated:OGD vs. non-treated:OGD	-0.06149	-0.3041 to 0.1811	No	ns	>0.9999
SB-treated:OGD vs. SB-treated:Ctr	-0.4089	-0.6516 to -0.1663	Yes	***	0.0007
Gv-treated:Ctr vs. SB-treated:Ctr	-0.1175	-0.3602 to 0.1251	No	ns	>0.9999

Gv-treated:OGD vs. SB-treated:Ctr	-0.1892	-0.4318 to 0.05342	No	ns	0.2212
Gv-treated:Ctr vs. SB-treated:OGD	0.2914	0.04878 to 0.5340	Yes	*	0.0134
Gv-treated:OGD vs. SB-treated:OGD	0.2197	-0.02291 to 0.4624	No	ns	0.0944
Gv-treated:OGD vs. Gv-treated:Ctr	-0.07169	-0.3143 to 0.1709	No	ns	>0.9999

CD206

24 h

Bonferroni's multiple comparisons test	Mean Diff.	95.00% CI of diff.	Below threshold?	Summary	Adjusted P Value
non-treated:OGD vs. non-treated:Ctr	-0.04239	-1.718 to 1.633	No	ns	>0.9999
SB-treated:Ctr vs. non-treated:Ctr	5.533	3.857 to 7.209	Yes	****	<0.0001
SB-treated:OGD vs. non-treated:Ctr	5.003	3.328 to 6.679	Yes	****	<0.0001
Gv-treated:Ctr vs. non-treated:Ctr	-0.7462	-2.422 to 0.9296	No	ns	>0.9999
Gv-treated:OGD vs. non-treated:Ctr	0.1537	-1.522 to 1.829	No	ns	>0.9999
SB-treated:Ctr vs. non-treated:OGD	5.575	3.899 to 7.251	Yes	****	<0.0001
SB-treated:OGD vs. non-treated:OGD	5.046	3.370 to 6.722	Yes	****	<0.0001
Gv-treated:Ctr vs. non-treated:OGD	-0.7038	-2.380 to 0.9720	No	ns	>0.9999
Gv-treated:OGD vs. non-treated:OGD	0.1961	-1.480 to 1.872	No	ns	>0.9999
SB-treated:OGD vs. SB-treated:Ctr	-0.5295	-2.205 to 1.146	No	ns	>0.9999
Gv-treated:Ctr vs. SB-treated:Ctr	-6.279	-7.955 to -4.603	Yes	****	<0.0001
Gv-treated:OGD vs. SB-treated:Ctr	-5.379	-7.055 to -3.703	Yes	****	<0.0001
Gv-treated:Ctr vs. SB-treated:OGD	-5.75	-7.425 to -4.074	Yes	****	<0.0001
Gv-treated:OGD vs. SB-treated:OGD	-4.85	-6.525 to -3.174	Yes	****	<0.0001
Gv-treated:OGD vs. Gv-treated:Ctr	0.8999	-0.7759 to 2.576	No	ns	>0.9999

72 h

Bonferroni's multiple	Mean Diff.	95.00% CI of	Below	Summary	Adjuste

comparisons test		diff.	threshold ?		d P Value
non-treated:OGD vs. non-treated:Ctr	-0.7274	-1.278 to -0.1770	Yes	**	0.0063
SB-treated:Ctr vs. non-treated:Ctr	-0.8166	-1.367 to -0.2662	Yes	**	0.0023
SB-treated:OGD vs. non-treated:Ctr	-0.7911	-1.341 to -0.2407	Yes	**	0.0031
Gv-treated:Ctr vs. non-treated:Ctr	1.989	1.439 to 2.540	Yes	****	<0.0001
Gv-treated:OGD vs. non-treated:Ctr	-0.9145	-1.465 to -0.3641	Yes	***	0.0008
SB-treated:Ctr vs. non-treated:OGD	-0.08924	-0.6396 to 0.4612	No	ns	>0.9999
SB-treated:OGD vs. non-treated:OGD	-0.06375	-0.6141 to 0.4866	No	ns	>0.9999
Gv-treated:Ctr vs. non-treated:OGD	2.717	2.166 to 3.267	Yes	****	<0.0001
Gv-treated:OGD vs. non-treated:OGD	-0.1872	-0.7376 to 0.3632	No	ns	>0.9999
SB-treated:OGD vs. SB-treated:Ctr	0.02549	-0.5249 to 0.5759	No	ns	>0.9999
Gv-treated:Ctr vs. SB-treated:Ctr	2.806	2.255 to 3.356	Yes	****	<0.0001
Gv-treated:OGD vs. SB-treated:Ctr	-0.09795	-0.6483 to 0.4524	No	ns	>0.9999
Gv-treated:Ctr vs. SB-treated:OGD	2.78	2.230 to 3.331	Yes	****	<0.0001
Gv-treated:OGD vs. SB-treated:OGD	-0.1234	-0.6738 to 0.4270	No	ns	>0.9999
Gv-treated:OGD vs. Gv-treated:Ctr	-2.904	-3.454 to -2.353	Yes	****	<0.0001

2. Flow cytometry analysis

CD11b+CD86+

24 h

Bonferroni's multiple comparisons test	Mean Diff.	95.00% CI of diff.	Below threshold?	Summary	Adjusted P Value
non treated:OGD vs. non treated:Ctr	-2.95	-6.776 to 0.8762	No	ns	0.2679
SB treated:Ctr vs. non treated	-0.2	-	No	ns	>0.9999

:Ctr		4.026 to 3.626			
SB treated:OGD vs. non treated:Ctr	0.125	-3.701 to 3.951	No	ns	>0.9999
Gv treated:Ctr vs. non treated:Ctr	-0.425	-4.251 to 3.401	No	ns	>0.9999
Gv treated:OGD vs. non treated:Ctr	-1.05	-4.876 to 2.776	No	ns	>0.9999
SB treated:Ctr vs. non treated:OGD	2.75	-1.076 to 6.576	No	ns	0.3871
SB treated:OGD vs. non treated:OGD	3.075	-0.7512 to 6.901	No	ns	0.2121
Gv treated:Ctr vs. non treated:OGD	2.525	-1.301 to 6.351	No	ns	0.5798
Gv treated:OGD vs. non treated:OGD	1.9	-1.926 to 5.726	No	ns	>0.9999
SB treated:OGD vs. SB treated:Ctr	0.325	-3.501 to 4.151	No	ns	>0.9999
Gv treated:Ctr vs. SB treated:Ctr	-0.225	-4.051 to 3.601	No	ns	>0.9999
Gv treated:OGD vs. SB treated:Ctr	-0.85	-4.676 to 2.976	No	ns	>0.9999
Gv treated:Ctr vs. SB treated:OGD	-0.55	-4.376 to 3.276	No	ns	>0.9999
Gv treated:OGD vs. SB treated:OGD	-1.175	-5.001 to 2.651	No	ns	>0.9999
Gv treated:OGD vs. Gv treated:Ctr	-0.625	-4.451 to 3.201	No	ns	>0.9999

72 h

Bonferroni's multiple comparisons test	Mean Diff.	95.00% CI of diff.	Below threshold?	Summary	Adjusted P Value
non treated:OGD vs. non treated:Ctr	1.95	-20.74 to 24.6	No	ns	>0.9999

		4			
SB treated:Ctr vs. non treated:Ctr	0.6	-22.09 to 23.29	No	ns	>0.9999
SB treated:OGD vs. non treated:Ctr	-5.25	-27.94 to 17.44	No	ns	>0.9999
Gv treated:Ctr vs. non treated:Ctr	0.625	-22.06 to 23.31	No	ns	>0.9999
Gv treated:OGD vs. non treated:Ctr	-8.675	-31.36 to 14.01	No	ns	>0.9999
SB treated:Ctr vs. non treated:OGD	-1.35	-24.04 to 21.34	No	ns	>0.9999
SB treated:OGD vs. non treated:OGD	-7.2	-29.89 to 15.49	No	ns	>0.9999
Gv treated:Ctr vs. non treated:OGD	-1.325	-24.01 to 21.36	No	ns	>0.9999
Gv treated:OGD vs. non treated:OGD	-10.63	-33.31 to 12.06	No	ns	>0.9999
SB treated:OGD vs. SB treated:Ctr	-5.85	-28.54 to 16.84	No	ns	>0.9999
Gv treated:Ctr vs. SB treated:Ctr	0.025	-22.66 to 22.71	No	ns	>0.9999
Gv treated:OGD vs. SB treated:Ctr	-9.275	-31.96 to 13.41	No	ns	>0.9999
Gv treated:Ctr vs. SB treated:OGD	5.875	-16.81 to 28.56	No	ns	>0.9999
Gv treated:OGD vs. SB treated:OGD	-3.425	-26.11 to 19.26	No	ns	>0.9999
Gv treated:OGD vs. Gv treated:Ctr	-9.3	-31.99 to 13.39	No	ns	>0.9999

CD11b+CD206+

24 h

Bonferroni's multiple comparisons test	Mean Diff.	95.00% CI of diff.	Below threshold?	Summary	Adjusted P Value

non treated:OGD vs. non treated:Ctr	0.025	-4.424 to 4.474	No	ns	>0.9999
SB treated:Ctr vs. non treated:Ctr	0.75	-3.699 to 5.199	No	ns	>0.9999
SB treated:OGD vs. non treated:Ctr	0.425	-4.024 to 4.874	No	ns	>0.9999
Gv treated:Ctr vs. non treated:Ctr	0.2	-4.249 to 4.649	No	ns	>0.9999
Gv treated:OGD vs. non treated:Ctr	-0.275	-4.724 to 4.174	No	ns	>0.9999
SB treated:Ctr vs. non treated:OGD	0.725	-3.724 to 5.174	No	ns	>0.9999
SB treated:OGD vs. non treated:OGD	0.4	-4.049 to 4.849	No	ns	>0.9999
Gv treated:Ctr vs. non treated:OGD	0.175	-4.274 to 4.624	No	ns	>0.9999
Gv treated:OGD vs. non treated:OGD	-0.3	-4.749 to 4.149	No	ns	>0.9999
SB treated:OGD vs. SB treated:Ctr	-0.325	-4.774 to 4.124	No	ns	>0.9999
Gv treated:Ctr vs. SB treated:Ctr	-0.55	-4.999 to 3.899	No	ns	>0.9999
Gv treated:OGD vs. SB treated:Ctr	-1.025	-5.474 to 3.424	No	ns	>0.9999
Gv treated:Ctr vs. SB treated:OGD	-0.225	-4.674 to 4.224	No	ns	>0.9999
Gv treated:OGD vs. SB treated:OGD	-0.7	-5.149 to 3.749	No	ns	>0.9999
Gv treated:OGD vs. Gv treated:Ctr	-0.475	-4.924 to 3.974	No	ns	>0.9999

<u>72 h</u>

Bonferroni's multiple comparisons test	Mean Diff.	95.00% CI of diff.	Below threshold?	Summary	Adjusted P Value

non treated:OGD vs. non treated:Ctr	-3.2	-17.58 to 11.18	No	ns	>0.9999
SB treated:Ctr vs. non treated:Ctr	6.9	-7.482 to 21.28	No	ns	>0.9999
SB treated:OGD vs. non treated:Ctr	7.9	-6.482 to 22.28	No	ns	>0.9999
Gv treated:Ctr vs. non treated:Ctr	-0.65	-15.03 to 13.73	No	ns	>0.9999
Gv treated:OGD vs. non treated:Ctr	-1.325	-15.71 to 13.06	No	ns	>0.9999
SB treated:Ctr vs. non treated:OGD	10.1	-4.282 to 24.48	No	ns	0.4339
SB treated:OGD vs. non treated:OGD	11.1	-3.282 to 25.48	No	ns	0.2664
Gv treated:Ctr vs. non treated:OGD	2.55	-11.83 to 16.93	No	ns	>0.9999
Gv treated:OGD vs. non treated:OGD	1.875	-12.51 to 16.26	No	ns	>0.9999
SB treated:OGD vs. SB treated:Ctr	1	-13.38 to 15.38	No	ns	>0.9999
Gv treated:Ctr vs. SB treated:Ctr	-7.55	-21.93 to 6.832	No	ns	>0.9999
Gv treated:OGD vs. SB treated:Ctr	-8.225	-22.61 to 6.157	No	ns	>0.9999
Gv treated:Ctr vs. SB treated:OGD	-8.55	-22.93 to 5.832	No	ns	0.8955
Gv treated:OGD vs. SB treated:OGD	-9.225	-23.61 to 5.157	No	ns	0.6567
Gv treated:OGD vs. Gv treated:Ctr	-0.675	-15.06 to 13.71	No	ns	>0.9999

CD86+CD206+

24 h

Bonferroni's multiple comparisons test	Mean Diff.	95.00% CI of diff.	Below threshold?	Summary	Adjusted P Value
non treated:OGD vs. non treated:Ctr	0.05	-4.219 to 4.319	No	ns	>0.9999
SB treated:Ctr vs. non treated:Ctr	0.375	-3.894 to 4.644	No	ns	>0.9999
SB treated:OGD vs. non treated:Ctr	0.325	-3.944 to 4.594	No	ns	>0.9999
Gv treated:Ctr vs. non treated:Ctr	-0.15	-4.419 to 4.119	No	ns	>0.9999
Gv treated:OGD vs. non treated:Ctr	-0.325	-4.594 to 3.944	No	ns	>0.9999
SB treated:Ctr vs. non treated:OGD	0.325	-3.944 to 4.594	No	ns	>0.9999
SB treated:OGD vs. non treated:OGD	0.275	-3.994 to 4.544	No	ns	>0.9999
Gv treated:Ctr vs. non treated:OGD	-0.2	-4.469 to 4.069	No	ns	>0.9999
Gv treated:OGD vs. non treated:OGD	-0.375	-4.644 to 3.894	No	ns	>0.9999
SB treated:OGD vs. SB treated:Ctr	-0.05	-4.319 to 4.219	No	ns	>0.9999
Gv treated:Ctr vs. SB treated:Ctr	-0.525	-4.794 to 3.744	No	ns	>0.9999
Gv treated:OGD vs. SB treated:Ctr	-0.7	-4.969 to 3.569	No	ns	>0.9999
Gv treated:Ctr vs. SB treated:OGD	-0.475	-4.744 to 3.794	No	ns	>0.9999
Gv treated:OGD vs. SB treated:OGD	-0.65	-4.919 to 3.619	No	ns	>0.9999
Gv treated:OGD vs. Gv treated:Ctr	-0.175	-4.444 to 4.094	No	ns	>0.9999

72 h

Bonferroni's multiple comparisons test	Mean diff.	95.00% CI of diff.	Below threshold?	Summary	Adjusted P Value
non treated:OGD vs. non treated:Ctr	-3.125	-17.87 to 11.62	No	ns	>0.9999
SB treated:Ctr vs. non treated:Ctr	6.175	-8.574 to 20.92	No	ns	>0.9999
SB treated:OGD vs. non treated:Ctr	8.825	-5.924 to 23.57	No	ns	0.8734
Gv treated:Ctr vs. non treated:Ctr	-0.425	-15.17 to 14.32	No	ns	>0.9999
Gv treated:OGD vs. non treated:Ctr	0.45	-14.30 to 15.20	No	ns	>0.9999
SB treated:Ctr vs. non treated:OGD	9.3	-5.449 to 24.05	No	ns	0.7063
SB treated:OGD vs. non treated:OGD	11.95	-2.799 to 26.70	No	ns	0.2024
Gv treated:Ctr vs. non treated:OGD	2.7	-12.05 to 17.45	No	ns	>0.9999
Gv treated:OGD vs. non treated:OGD	3.575	-11.17 to 18.32	No	ns	>0.9999
SB treated:OGD vs. SB treated:Ctr	2.65	-12.10 to 17.40	No	ns	>0.9999
Gv treated:Ctr vs. SB treated:Ctr	-6.6	-21.35 to 8.149	No	ns	>0.9999
Gv treated:OGD vs. SB treated:Ctr	-5.725	-20.47 to 9.024	No	ns	>0.9999
Gv treated:Ctr vs. SB treated:OGD	-9.25	-24.00 to 5.499	No	ns	0.7224
Gv treated:OGD vs. SB treated:OGD	-8.375	-23.12 to 6.374	No	ns	>0.9999
Gv treated:OGD vs. Gv treated:Ctr	0.875	-13.87 to 15.62	No	ns	>0.9999

3. Western Blot analysis

p-AKT

SB-treated

24 h

Bonferroni's multiple comparisons test	Mean diff.	95.00% CI of diff.	Below threshold?	Summary	Adjusted P Value
non treated:OGD vs. non treated:Control	-25.43	-83.83 to 32.97	No	ns	>0.9999
SB-treated:Control vs. non treated:Control	12.07	-46.33 to 70.47	No	ns	>0.9999
SB-treated:OGD vs. non treated:Control	44.17	-14.23 to 102.6	No	ns	0.2474
SB-treated:Control vs. non treated:OGD	37.5	-20.90 to 95.90	No	ns	0.4822
SB-treated:OGD vs. non treated:OGD	69.6	11.20 to 128.0	Yes	*	0.0124
SB-treated:OGD vs. SB-treated:Control	32.1	-26.30 to 90.50	No	ns	0.792

72 h

Bonferroni's multiple comparisons test	Predicted (LS) Mean diff.	95.00% CI of diff.	Below threshold?	Summary	Adjusted P Value
non treated:OGD vs. non treated:Control	27.51	-48.44 to 103.5	No	ns	>0.9999
SB-treated:Control vs. non treated:Control	58.24	-28.35 to 144.8	No	ns	0.3927
SB-treated:OGD vs. non treated:Control	179.3	103.3 to 255.2	Yes	****	<0.0001
SB-treated:Control vs. non treated:OGD	30.73	-55.86 to 117.3	No	ns	>0.9999
SB-treated:OGD vs. non treated:OGD	151.8	75.82 to 227.7	Yes	****	<0.0001
SB-treated:OGD vs. SB-treated:Control	121	34.45 to 207.6	Yes	**	0.0029

Gv-treated

<u>24 h</u>

Bonferroni's multiple compariso ns test	Mean d iff.	95.00% CI of diff.	Below thresh old?	Summ ary	Adjusted P V alue
non treated:OGD vs. non treate d:Control	-39.14	-60.32 to - 17.96	Yes	****	<0.0001
Gv- treated:Control vs. non treated:C ontrol	-11.82	- 33.00 to 9.36 0	No	ns	0.7582
Gv- treated:OGD vs. non treated:Con trol	-21.07	- 42.25 to 0.11 00	No	ns	0.0518
Gv- treated:Control vs. non treated: OGD	27.32	6.140 to 48.5 0	Yes	**	0.0059
Gv- treated:OGD vs. non treated:OG D	18.07	- 3.110 to 39.2 5	No	ns	0.1345
Gv-treated:OGD vs. Gv- treated:Control	-9.25	- 30.43 to 11.9 3	No	ns	>0.9999

<u>72 h</u>

Bonferroni's multiple compariso ns test	Mean d iff.	95.00% CI of diff.	Below thresh old?	Summ ary	Adjusted P V alue
non treated:OGD vs. non treate d:Control	22.11	- 41.49 to 85.7 1	No	ns	>0.9999
Gv- treated:Control vs. non treated:C ontrol	50.15	- 13.45 to 113. 8	No	ns	0.1909
Gv- treated:OGD vs. non treated:Con trol	14.4	- 49.20 to 78.0 0	No	ns	>0.9999
Gv- treated:Control vs. non treated: OGD	28.04	- 35.56 to 91.6 4	No	ns	>0.9999
Gv- treated:OGD vs. non treated:OG D	-7.71	- 71.31 to 55.8 9	No	ns	>0.9999
Gv-treated:OGD vs. Gv- treated:Control	-35.75	- 99.35 to 27.8 5	No	ns	0.6932

AKT total

SB-treated

24 h

Bonferroni's multiple comparisons test	Mean diff.	95.00% CI of diff.	Below threshold?	Summary	Adjusted P Value
non treated:OGD vs. non treated:Control	-8.26	-25.86 to 9.342	No	ns	>0.9999
SB-treated:Control vs. non treated:Control	-8.16	-25.76 to 9.442	No	ns	>0.9999
SB-treated:OGD vs. non treated:Control	-3.67	-21.27 to 13.93	No	ns	>0.9999
SB-treated:Control vs. non treated:OGD	0.1	-17.50 to 17.70	No	ns	>0.9999
SB-treated:OGD vs. non treated:OGD	4.59	-13.01 to 22.19	No	ns	>0.9999
SB-treated:OGD vs. SB-treated:Control	4.49	-13.11 to 22.09	No	ns	>0.9999

72 h

Bonferroni's multiple comparisons test	Mean diff.	95.00% CI of diff.	Below threshold?	Summary	Adjusted P Value
non treated:OGD vs. non treated:Control	1.2	-8.321 to 10.72	No	ns	>0.9999
SB-treated:Control vs. non treated:Control	-1.44	-10.96 to 8.081	No	ns	>0.9999
SB-treated:OGD vs. non treated:Control	-5.01	-14.53 to 4.511	No	ns	0.798
SB-treated:Control vs. non treated:OGD	-2.64	-12.16 to 6.881	No	ns	>0.9999
SB-treated:OGD vs. non treated:OGD	-6.21	-15.73 to 3.311	No	ns	0.4043
SB-treated:OGD vs. SB-treated:Control	-3.57	-13.09 to 5.951	No	ns	>0.9999

Gv-treated

24 h

Bonferroni's multiple comparisons test	Mean diff.	95.00% CI of diff.	Below threshold?	Summary	Adjusted P Value
non treated:OGD vs. non treated:Control	-10.32	-21.92 to 1.275	No	ns	0.1057
Giv-treated:Control vs. non treated:Control	3.39	-8.205 to 14.99	No	ns	>0.9999
Giv-treated:OGD vs. non treated:Control	-10.35	-21.95 to 1.245	No	ns	0.1039
Giv-treated:Control vs. non treated: OGD	13.71	2.115 to 25.31	Yes	*	0.0134
Giv-treated:OGD vs. non treated:OGD	-0.03	-11.63 to 11.57	No	ns	>0.9999
Giv-treated:OGD vs. Giv-treated:Control	-13.74	-25.34 to -2.145	Yes	*	0.0131

72 h

Bonferroni's multiple comparisons test	Mean diff.	95.00% CI of diff.	Below threshold?	Summary	Adjusted P Value
non treated:OGD vs. non treated:Control	-8.3	-25.11 to 8.515	No	ns	>0.9999
Giv-treated:Control vs. non treated:Control	4.46	-12.35 to 21.27	No	ns	>0.9999
Giv-treated:OGD vs. non treated:Control	-1.61	-18.42 to 15.20	No	ns	>0.9999
Giv-treated:Control vs. non treated: OGD	12.76	-4.055 to 29.57	No	ns	0.2349
Giv-treated:OGD vs. non treated:OGD	6.69	-10.12 to 23.50	No	ns	>0.9999
Giv-treated:OGD vs. Giv-treated:Control	-6.07	-22.88 to 10.74	No	ns	>0.9999

p-ERK

SB-treated

24 h

Bonferroni's multiple comparisons test	Mean diff.	95.00% CI of diff.	Below threshold?	Summary	Adjusted P Value
non treated:OGD vs. non treated:Control	-10.13	-34.12 to 13.86	No	ns	>0.9999
SB-treated:Control vs. non treated:Control	-13.41	-37.40 to 10.58	No	ns	0.7829
SB-treated:OGD vs. non treated:Control	-15.16	-39.15 to 8.826	No	ns	0.5303
SB-treated:Control vs. non treated: OGD	-3.28	-27.27 to 20.71	No	ns	>0.9999
SB-treated:OGD vs. non treated:OGD	-5.03	-29.02 to 18.96	No	ns	>0.9999
SB-treated:OGD vs. SB-treated:Control	-1.75	-25.74 to 22.24	No	ns	>0.9999

72 h

Bonferroni's multiple comparisons test	Mean diff.	95.00% CI of diff.	Below threshold?	Summary	Adjusted P Value
non treated:OGD vs. non treated:Control	2.31	-25.09 to 29.71	No	ns	>0.9999
SB-treated:Control vs. non treated:Control	-0.37	-27.77 to 27.03	No	ns	>0.9999
SB-treated:OGD vs. non treated:Control	-7.1	-34.50 to 20.30	No	ns	>0.9999
SB-treated:Control vs. non treated: OGD	-2.68	-30.08 to 24.72	No	ns	>0.9999
SB-treated:OGD vs. non treated:OGD	-9.41	-36.81 to 17.99	No	ns	>0.9999
SB-treated:OGD vs. SB-treated:Control	-6.73	-34.13 to 20.67	No	ns	>0.9999

Gv-teated

24 h

Bonferroni's multiple comparisons test	Mean diff.	95.00% CI of diff.	Below threshold?	Summary	Adjusted P Value
non treated:OGD vs. non treated:Control	-7.91	-26.01 to 10.19	No	ns	>0.9999
Giv-treated:Control vs. non treated:Control	-4.9	-23.00 to 13.20	No	ns	>0.9999
Giv-treated:OGD vs. non treated:Control	5.8	-12.30 to 23.90	No	ns	>0.9999
Giv-treated:Control vs. non treated: OGD	3.01	-15.09 to 21.11	No	ns	>0.9999
Giv-treated:OGD vs. non treated:OGD	13.71	-4.392 to 31.81	No	ns	0.2586
Giv-treated:OGD vs. Giv-treated:Control	10.7	-7.402 to 28.80	No	ns	0.6722

72 h

Bonferroni's multiple comparisons test	Mean diff.	95.00% CI of diff.	Below threshold?	Summary	Adjusted P Value
non treated:OGD vs. non treated:Control	0.1	-22.20 to 22.40	No	ns	>0.9999
Giv-treated:Control vs. non treated:Control	6.79	-15.51 to 29.09	No	ns	>0.9999
Giv-treated:OGD vs. non treated:Control	-10.13	-32.43 to 12.17	No	ns	>0.9999
Giv-treated:Control vs. non treated: OGD	6.69	-15.61 to 28.99	No	ns	>0.9999
Giv-treated:OGD vs. non treated:OGD	-10.23	-32.53 to 12.07	No	ns	>0.9999
Giv-treated:OGD vs. Giv-treated:Control	-16.92	-39.22 to 5.381	No	ns	0.2511

ERK

SB-treated

<u>24 h</u>

Bonferroni's multiple comparisons test	Mean diff.	95.00% CI of diff.	Below threshold?	Summary	Adjusted P Value
non treated:OGD vs. non treated:Control	-2.81	-24.22 to 18.60	No	ns	>0.9999
SB-treated:Control vs. non treated:Control	-5.61	-27.02 to 15.80	No	ns	>0.9999
SB-treated:OGD vs. non treated:Control	13.72	-7.690 to 35.13	No	ns	0.5081
SB-treated:Control vs. non treated: OGD	-2.8	-24.21 to 18.61	No	ns	>0.9999
SB-treated:OGD vs. non treated:OGD	16.53	-4.880 to 37.94	No	ns	0.234
SB-treated:OGD vs. SB-treated:Control	19.33	-2.080 to 40.74	No	ns	0.0994

<u>72 h</u>

Bonferroni's multiple comparisons test	Mean diff.	95.00% CI of diff.	Below threshold?	Summary	Adjusted P Value
non treated:OGD vs. non treated:Control	-8.93	-50.42 to 32.56	No	ns	>0.9999
SB-treated:Control vs. non treated:Control	19.42	-22.07 to 60.91	No	ns	>0.9999
SB-treated:OGD vs. non treated:Control	33.87	-7.621 to 75.36	No	ns	0.168
SB-treated:Control vs. non treated: OGD	28.35	-13.14 to 69.84	No	ns	0.3752
SB-treated:OGD vs. non treated:OGD	42.8	1.309 to 84.29	Yes	*	0.0402
SB-treated:OGD vs. SB-treated:Control	14.45	-27.04 to 55.9	No	ns	>0.9999

		4			

Gv-treated

24 h

Bonferroni's multiple comparisons test	Mean diff.	95.00% CI of diff.	Below threshold?	Summary	Adjusted P Value
non treated:OGD vs. non treated:Control	1.97	-14.47 to 18.41	No	ns	>0.9999
Giv-treated:Control vs. non treated:Control	5.07	-11.37 to 21.51	No	ns	>0.9999
Giv-treated:OGD vs. non treated:Control	-0.21	-16.65 to 16.23	No	ns	>0.9999
Giv-treated:Control vs. non treated: OGD	3.1	-13.34 to 19.54	No	ns	>0.9999
Giv-treated:OGD vs. non treated:OGD	-2.18	-18.62 to 14.26	No	ns	>0.9999
Giv-treated:OGD vs. Giv-treated:Control	-5.28	-21.72 to 11.16	No	ns	>0.9999

72 h

Bonferroni's multiple comparisons test	Mean diff.	95.00% CI of diff.	Below threshold?	Summary	Adjusted P Value
non treated:OGD vs. non treated:Control	-2.77	-20.52 to 14.98	No	ns	>0.9999
Giv-treated:Control vs. non treated:Control	0.72	-17.03 to 18.47	No	ns	>0.9999
Giv-treated:OGD vs. non treated:Control	6.71	-11.04 to 24.46	No	ns	>0.9999
Giv-treated:Control vs. non treated: OGD	3.49	-14.26 to 21.24	No	ns	>0.9999
Giv-treated:OGD vs. non treated:OGD	9.48	-8.274 to 27.23	No	ns	0.8767
Giv-treated:OGD vs. Giv-treated:Control	5.99	-11.76 to 23.7	No	ns	>0.9999

		4			

4. ELISA analysis

p-AKT

24 h

Bonferroni's multiple comparisons test	Mean Diff.	95.00% CI of diff.	Below threshold ?	Summary	Adjusted P Value
non treated:OGD vs. non treated:Control	-35.05	-79.30 to 9.198	No	ns	0.2035
SB-treated:Control vs. non treated:Control	10.5	-33.75 to 54.74	No	ns	>0.9999
SB-treated:OGD vs. non treated:Control	52.27	8.025 to 96.52	Yes	*	0.0152
Gv-treated:Control vs. non treated:Control	15.11	-29.14 to 59.36	No	ns	>0.9999
Gv-treated:OGD vs. non treated:Control	54.58	10.34 to 98.83	Yes	*	0.0109
SB-treated:Control vs. non treated:OGD	45.55	1.298 to 89.79	Yes	*	0.0411
SB-treated:OGD vs. non treated:OGD	87.32	43.07 to 131.6	Yes	***	0.0002
Gv-treated:Control vs. non treated:OGD	50.16	5.910 to 94.41	Yes	*	0.0207
Gv-treated:OGD vs. non treated:OGD	89.63	45.38 to 133.9	Yes	***	0.0001
SB-treated:OGD vs. SB-treated:Control	41.78	-2.471 to 86.03	No	ns	0.0727
Gv-treated:Control vs. SB-treated:Control	4.613	-39.64 to 48.86	No	ns	>0.9999
Gv-treated:OGD vs. SB-treated:Control	44.09	-0.1610 to 88.34	No	ns	0.0512
Gv-treated:Control vs. SB-treated:OGD	-37.16	-81.41 to 7.084	No	ns	0.1472
Gv-treated:OGD vs. SB-treated:OGD	2.31	-41.94 to 46.56	No	ns	>0.9999
Gv-treated:OGD vs. Gv-treated:Control	39.47	-4.774 to 83.72	No	ns	0.1033

72 h

Bonferroni's multiple comparisons test	Mean Diff.	95.00% CI of diff.	Below threshold ?	Summary	Adjusted P Value
non treated:OGD vs. non	88.06	20.35 to	Yes	**	0.0071

treated:Control		155.8			
SB-treated:Control vs. non treated:Control	19	-48.71 to 86.71	No	ns	>0.9999
SB-treated:OGD vs. non treated:Control	258.8	191.0 to 326.5	Yes	****	<0.0001
Gv-treated:Control vs. non treated:Control	58.47	-9.237 to 126.2	No	ns	0.1254
Gv-treated:OGD vs. non treated:Control	50.63	-17.08 to 118.3	No	ns	0.2748
SB-treated:Control vs. non treated:OGD	-69.06	-136.8 to -1.347	Yes	*	0.0438
SB-treated:OGD vs. non treated:OGD	170.7	103.0 to 238.4	Yes	****	<0.0001
Gv-treated:Control vs. non treated:OGD	-29.59	-97.30 to 38.12	No	ns	>0.9999
Gv-treated:OGD vs. non treated:OGD	-37.43	-105.1 to 30.28	No	ns	0.9993
SB-treated:OGD vs. SB-treated:Control	239.8	172.0 to 307.5	Yes	****	<0.0001
Gv-treated:Control vs. SB-treated:Control	39.47	-28.24 to 107.2	No	ns	0.8225
Gv-treated:OGD vs. SB-treated:Control	31.63	-36.08 to 99.34	No	ns	>0.9999
Gv-treated:Control vs. SB-treated:OGD	-200.3	-268.0 to -132.6	Yes	****	<0.0001
Gv-treated:OGD vs. SB-treated:OGD	-208.1	-275.8 to -140.4	Yes	****	<0.0001
Gv-treated:OGD vs. Gv-treated:Control	-7.845	-75.56 to 59.87	No	ns	>0.9999

AKT total

<u>24 h</u>

Bonferroni's multiple comparisons test	Mean Diff.	95.00% CI of diff.	Below threshold?	Summary	Adjusted P Value
non treated:OGD vs. non treated:Control	-25.66	-37.58 to -13.73	Yes	****	<0.0001
SB-treated:Control vs. non treated:Control	-14.91	-26.83 to -2.985	Yes	**	0.0098
SB-treated:OGD vs. non treated:Control	-21.98	-33.90 to -10.05	Yes	***	0.0003
Gv-treated:Control vs. non treated:Control	-6.387	-18.31 to 5.537	No	ns	>0.9999
Gv-treated:OGD vs. non treated:Control	-60.46	-72.38 to -48.53	Yes	****	<0.0001
SB-treated:Control vs. non treated:OGD	10.75	-1.176 to 22.67	No	ns	0.0971

SB-treated:OGD vs. non treated:OGD	3.679	-8.245 to 15.60	No	ns	>0.9999
Gv-treated:Control vs. non treated:OGD	19.27	7.345 to 31.19	Yes	**	0.0011
Gv-treated:OGD vs. non treated:OGD	-34.8	-46.72 to -22.88	Yes	****	<0.0001
SB-treated:OGD vs. SB-treated:Control	-7.068	-18.99 to 4.855	No	ns	0.7714
Gv-treated:Control vs. SB-treated:Control	8.521	-3.402 to 20.45	No	ns	0.3435
Gv-treated:OGD vs. SB-treated:Control	-45.55	-57.47 to -33.62	Yes	****	<0.0001
Gv-treated:Control vs. SB-treated:OGD	15.59	3.666 to 27.51	Yes	**	0.0068
Gv-treated:OGD vs. SB-treated:OGD	-38.48	-50.40 to -26.55	Yes	****	<0.0001
Gv-treated:OGD vs. Gv-treated:Control	-54.07	-65.99 to -42.14	Yes	****	<0.0001

72 h

Bonferroni's multiple comparisons test	Mean Diff.	95.00% CI of diff.	Below threshold?	Summary	Adjusted P Value
non treated:OGD vs. non treated:Control	0.8527	-18.35 to 20.05	No	ns	>0.9999
SB-treated:Control vs. non treated:Control	-33.01	-52.21 to -13.81	Yes	***	0.0006
SB-treated:OGD vs. non treated:Control	-7.672	-26.87 to 11.53	No	ns	>0.9999
Gv-treated:Control vs. non treated:Control	-25.01	-44.21 to -5.814	Yes	**	0.007
Gv-treated:OGD vs. non treated:Control	-4.419	-23.62 to 14.78	No	ns	>0.9999
SB-treated:Control vs. non treated:OGD	-33.86	-53.06 to -14.66	Yes	***	0.0005
SB-treated:OGD vs. non treated:OGD	-8.525	-27.72 to 10.67	No	ns	>0.9999
Gv-treated:Control vs. non treated:OGD	-25.87	-45.06 to -6.666	Yes	**	0.0053
Gv-treated:OGD vs. non treated:OGD	-5.272	-24.47 to 13.93	No	ns	>0.9999
SB-treated:OGD vs. SB-treated:Control	25.33	6.136 to 44.53	Yes	**	0.0063
Gv-treated:Control vs. SB-treated:Control	7.994	-11.20 to 27.19	No	ns	>0.9999
Gv-treated:OGD vs. SB-treated:Control	28.59	9.389 to 47.79	Yes	**	0.0023
Gv-treated:Control vs. SB-treated:OGD	-17.34	-36.54 to	No	ns	0.0959

		1.859			
Gv-treated:OGD vs. SB-treated:OGD	3.253	-15.95 to 22.45	No	ns	>0.9999
Gv-treated:OGD vs. Gv-treated:Control	20.59	1.395 to 39.79	Yes	*	0.0309

p-ERK

24 h

Bonferroni's multiple comparisons test	Mean Diff.	95.00% CI of diff.	Below threshold ?	Summary	Adjusted P Value
non treated:OGD vs. non treated:Control	-10.59	-24.77 to 3.582	No	ns	0.2756
SB-treated:Control vs. non treated:Control	-13.03	-27.21 to 1.142	No	ns	0.0859
SB-treated:OGD vs. non treated:Control	-23.11	-37.29 to -8.936	Yes	**	0.001
Gv-treated:Control vs. non treated:Control	-15.05	-29.22 to -0.8708	Yes	*	0.0332
Gv-treated:OGD vs. non treated:Control	-14.3	-28.48 to -0.1273	Yes	*	0.0471
SB-treated:Control vs. non treated:OGD	-2.44	-16.62 to 11.74	No	ns	>0.9999
SB-treated:OGD vs. non treated:OGD	-12.52	-26.69 to 1.658	No	ns	0.1099
Gv-treated:Control vs. non treated:OGD	-4.453	-18.63 to 9.723	No	ns	>0.9999
Gv-treated:OGD vs. non treated:OGD	-3.709	-17.88 to 10.47	No	ns	>0.9999
SB-treated:OGD vs. SB-treated:Control	-10.08	-24.25 to 4.097	No	ns	0.3521
Gv-treated:Control vs. SB-treated:Control	-2.013	-16.19 to 12.16	No	ns	>0.9999
Gv-treated:OGD vs. SB-treated:Control	-1.27	-15.44 to 12.91	No	ns	>0.9999
Gv-treated:Control vs. SB-treated:OGD	8.065	-6.110 to 22.24	No	ns	0.9007
Gv-treated:OGD vs. SB-treated:OGD	8.808	-5.367 to 22.98	No	ns	0.6396
Gv-treated:OGD vs. Gv-treated:Control	0.7435	-13.43 to 14.92	No	ns	>0.9999

72 h

Bonferroni's multiple comparisons test	Mean Diff.	95.00% CI of diff.	Below threshold ?	Summary	Adjusted P Value

non treated:OGD vs. non treated:Control	12.05	-13.78 to 37.89	No	ns	>0.9999
SB-treated:Control vs. non treated:Control	22.19	-3.645 to 48.03	No	ns	0.1294
SB-treated:OGD vs. non treated:Control	2.72	-23.12 to 28.56	No	ns	>0.9999
Gv-treated:Control vs. non treated:Control	0.7128	-25.12 to 26.55	No	ns	>0.9999
Gv-treated:OGD vs. non treated:Control	1.933	-23.90 to 27.77	No	ns	>0.9999
SB-treated:Control vs. non treated:OGD	10.14	-15.70 to 35.98	No	ns	>0.9999
SB-treated:OGD vs. non treated:OGD	-9.33	-35.17 to 16.51	No	ns	>0.9999
Gv-treated:Control vs. non treated:OGD	-11.34	-37.17 to 14.50	No	ns	>0.9999
Gv-treated:OGD vs. non treated:OGD	-10.12	-35.95 to 15.72	No	ns	>0.9999
SB-treated:OGD vs. SB-treated:Control	-19.47	-45.31 to 6.365	No	ns	0.2641
Gv-treated:Control vs. SB-treated:Control	-21.48	-47.31 to 4.358	No	ns	0.156
Gv-treated:OGD vs. SB-treated:Control	-20.26	-46.09 to 5.578	No	ns	0.2149
Gv-treated:Control vs. SB-treated:OGD	-2.007	-27.84 to 23.83	No	ns	>0.9999
Gv-treated:OGD vs. SB-treated:OGD	-0.7869	-26.62 to 25.05	No	ns	>0.9999
Gv-treated:OGD vs. Gv-treated:Control	1.22	-24.61 to 27.06	No	ns	>0.9999

ERK total

24 h

Bonferroni's multiple comparisons test	Mean Diff.	95.00% CI of diff.	Below threshold ?	Summar y	Adjuste d P Value
non treated:OGD vs. non treated:Control	-24.06	-36.10 to -12.02	Yes	***	0.0001
SB-treated:Control vs. non treated:Control	-6.988	-19.02 to 5.047	No	ns	0.8351
SB-treated:OGD vs. non treated:Control	-14.79	-26.83 to -2.757	Yes	*	0.0112
Gv-treated:Control vs. non treated:Control	-3.709	-15.74 to 8.326	No	ns	>0.9999
Gv-treated:OGD vs. non treated:Control	-19.49	-31.53 to -7.457	Yes	**	0.0011
SB-treated:Control vs. non	17.07	5.037 to	Yes	**	0.0035

treated:OGD		29.11			
SB-treated:OGD vs. non treated:OGD	9.268	-2.767 to 21.30	No	ns	0.2363
Gv-treated:Control vs. non treated:OGD	20.35	8.316 to 32.39	Yes	***	0.0007
Gv-treated:OGD vs. non treated:OGD	4.568	-7.467 to 16.60	No	ns	>0.9999
SB-treated:OGD vs. SB-treated:Control	-7.804	-19.84 to 4.231	No	ns	0.5348
Gv-treated:Control vs. SB-treated:Control	3.279	-8.757 to 15.31	No	ns	>0.9999
Gv-treated:OGD vs. SB-treated:Control	-12.5	-24.54 to -0.4689	Yes	*	0.0386
Gv-treated:Control vs. SB-treated:OGD	11.08	-0.9524 to 23.12	No	ns	0.0851
Gv-treated:OGD vs. SB-treated:OGD	-4.7	-16.74 to 7.335	No	ns	>0.9999
Gv-treated:OGD vs. Gv-treated:Control	-15.78	-27.82 to -3.748	Yes	**	0.0067

72 h

Bonferroni's multiple comparisons test	Mean Diff.	95.00% CI of diff.	Below threshold?	Summary	Adjusted P Value
non treated:OGD vs. non treated:Control	13.4	-5.927 to 32.72	No	ns	0.3964
SB-treated:Control vs. non treated:Control	14.6	-4.729 to 33.92	No	ns	0.2612
SB-treated:OGD vs. non treated:Control	-4.386	-23.71 to 14.94	No	ns	>0.9999
Gv-treated:Control vs. non treated:Control	13.53	-5.792 to 32.86	No	ns	0.3783
Gv-treated:OGD vs. non treated:Control	6.22	-13.11 to 25.55	No	ns	>0.9999
SB-treated:Control vs. non treated:OGD	1.197	-18.13 to 20.52	No	ns	>0.9999
SB-treated:OGD vs. non treated:OGD	-17.79	-37.11 to 1.540	No	ns	0.0854
Gv-treated:Control vs. non treated:OGD	0.1348	-19.19 to 19.46	No	ns	>0.9999
Gv-treated:OGD vs. non treated:OGD	-7.179	-26.50 to 12.15	No	ns	>0.9999
SB-treated:OGD vs. SB-treated:Control	-18.98	-38.31 to 0.3432	No	ns	0.0563
Gv-treated:Control vs. SB-treated:Control	-1.062	-20.39 to 18.26	No	ns	>0.9999
Gv-treated:OGD vs. SB-treated:Control	-8.376	-27.70 to 10.95	No	ns	>0.9999

Gv-treated:Control vs. SB-treated:OGD	17.92	-1.405 to 37.25	No	ns	0.0815
Gv-treated:OGD vs. SB-treated:OGD	10.61	-8.719 to 29.93	No	ns	>0.9999
Gv-treated:OGD vs. Gv-treated:Control	-7.314	-26.64 to 12.01	No	ns	>0.9999
